# Supplementary material for: Ward-based Goal-Directed Fluid Therapy (GDFT) in Acute Pancreatitis (GAP) trial: study protocol for a feasibility randomised controlled trial
Source: BMJ Open. 2019 Oct 9;9(10):e028783. doi: 10.1136/bmjopen-2018-028783 (PMC6797248; doi:10.1136/bmjopen-2018-028783)
Supplement: Author's manuscript [file bmjopen-2018-028783.draft_revisions.pdf]

BMJ Open

BMJ Open is committed to open peer review. As part of this commitment we make the peer review history of every article we publish publicly available.

When an article is published we post the peer reviewers' comments and the authors' responses online. We also post the versions of the paper that were used during peer review. These are the versions that the peer review comments apply to.

The versions of the paper that follow are the versions that were submitted during the peer review process. They are not the versions of record or the final published versions. They should not be cited or distributed as the published version of this manuscript.

BMJ Open is an open access journal and the full, final, typeset and author-corrected version of record of the manuscript is available on our site with no access controls, subscription charges or pay-per-view fees (http://bmjopen.bmj.com).

If you have any questions on BMJ Open's open peer review process please email info.bmjopen@bmj.com

BMJ Open

Ward based Goal-Directed Fluid Therapy (GDFT) in Acute Pancreatitis (GAP trial): study protocol for a feasibility randomised controlled trial

Journal:	*BMJ Open*
Manuscript ID	bmjopen-2018-028783
Article Type:	Protocol
Date Submitted by the Author:	22-Dec-2018
Complete List of Authors:	Froghi, Farid; University College London, Division of Surgery & Interventional Science; Royal Free Hospital, HPB and Liver Transplantation Surgery<br>Soggiu, Fiammetta; Royal Free Hospital, HPB and Liver Transplantation Surgery<br>Ricciardi, Federico; University College London, Statistical Sciences<br>Gurusamy, Kurinchi; University College London, Division of Surgery & Interventional Sciences<br>Martin, Daniel S.; University College London, Division of Surgery & Interventional Sciences; Royal Free Hospital, Critical Care Unit<br>Singh, Jeshika; PHMR Ltd, Health Economics<br>Siddique, Sulman; University College London, Division of Surgery & Interventional Sciences<br>Eastgate, Christine; Royal Free Hospital, Critical Care Unit<br>Ciaponi, Maria; Royal Free Hospital, Critical Care Unit<br>McNeil, Margaret; Royal Free Hospital, Critical Care Unit<br>Filipe, Helder; Royal Free Hospital, Critical Care Unit<br>Schwalowsky-Monks, Otto; Royal Free Hospital, Critical Care Unit<br>Asis, Gretchelle; Royal Free Hospital, Critical Care Unit<br>Varcada, Massimo; Royal Free Hospital, General and Emergency Surgery<br>Davidson, Brian; University College London, Division of Surgery & Interventional Sciences; Royal Free Hospital, HPB and Liver Transplantation Surgery
Keywords:	Acute Pancreatitis, Goal-Directed Fluid Therapy, Fluid Therapy, Cardiac Output

SCHOLARONE™
Manuscripts

Ward based Goal-Directed Fluid Therapy (GDFT) in Acute Pancreatitis (GAP trial): study protocol for a feasibility randomised controlled trial

Farid Froghi[1], Fiammetta Soggiu[1], Federico Ricciardi[5], Kurinchi Gurusamy[1], Daniel Martin[1,2], Jeshika Singh[4], Sulman Siddique[1], Christine Eastgate[2], Maria Ciaponi[2], Margaret McNeil[2], Helder Filipe[2], Otto Schwalowsky-Monks[2], Gretchelle Asis[2], Massimo Varcada[1,3], Brian Davidson[1]

[1]UCL Division of Surgery and Interventional Science, Royal Free Hospital, London

[2]Critical Care Unit, Royal Free Hospital, London

[3]General and Emergency Surgery Department, Royal Free Hospital, London

[4]PHMR, Health Economics, Pricing & Reimbursement, London, United Kingdom

[5]Department of Statistical Science, University College London, London

Corresponding author: Mr Farid Froghi
UCL Division of Surgery & Interventional Sciences
9th Floor, Royal Free Hospital
Pond Street
London NW3 2QG

Email: farid.froghi@nhs.net

Tel: 020 7794 0500

For peer review only

Abstract

Background: Acute pancreatitis is an inflammatory disease of the pancreas with high risk of developing multiorgan failure and death. There are no effective pharmacological interventions used in current clinical practice. Maintaining fluid and electrolyte balance, particularly over the first 48 hours of onset, is the mainstay of supportive management. Goal directed fluid therapy (GDFT) has been shown to decrease morbidity and mortality in surgical conditions with systemic inflammatory response. There is currently no randomized controlled trial (RCT) investigating the role of GDFT based on cardiac output parameters in patients with acute pancreatitis in the ward setting. A feasibility trial was designed to determine patient and clinician support for recruitment into a RCT of ward-based GDFT in acute pancreatitis, adherence to a GDFT protocol, safety, participant withdrawal, and to determine appropriate endpoints for a subsequent larger trial to evaluate efficacy.

Methods and Analysis: The GDFT in Acute Pancreatitis (GAP) trial is a prospective two-centre, feasibility RCT. Eligible adults admitted with new onset of acute pancreatitis will be enrolled and randomised into ward-based GDFT (n=25) or standard fluid therapy (n=25) within 6 hours from the diagnosis and continuing for the following 48 hours. Cardiac output parameters will be monitored with a non-invasive device (Cheetah NICOM ®; Cheetah Medical Ltd, Maidenhead, Berkshire, UK). The intervention will consist of a protocoled GDFT approach consisting of stroke volume optimisation with crystalloid fluid boluses, while the control group will receive standard care fluid therapy as advised by the clinical team. The primary endpoint is feasibility. Secondary endpoints will include safety of the intervention, complications, mortality, admission to intensive care unit, cost and quality of life.

Funding and Ethics: This study is funded by NIHR RfPB and has been approved by the London Central Research Ethics Committee (17/LO/1235, project-ID: 221872).

Trial registration: ISRCTN 36077283 (26-03-2018)

Keywords:

Fluid therapy, Acute Pancreatitis, Cardiac Output, Goal-directed Fluid Therapy

Strengths and limitations of this study

- Adequate fluid therapy is a major determinant of outcomes in Acute Pancreatitis, however, there is no consensus on the rate, type and volume of fluids to be administered. GDFT based on SV optimisation has been used after major surgery with a reduction in complications, however, delivering this intervention on the ward has been challenging without invasive monitoring.
- Novel development of NICOM technologies allows GDFT to be delivered in the ward based on haemodynamic parameters. The GAP trial is the first GDFT randomised trial in acute pancreatitis using non-invasive cardiac output monitoring in the ward setting for SV optimisation.
- The golden period for intervention in acute pancreatitis is in the early phase of disease to prevent progression to severe inflammatory processes and complications. A major challenge in this trial is recruitment and delivering the intervention in the early hours of unplanned admission which is shared across trials in emergency care.

For peer review only

Background

Acute pancreatitis is a sudden onset inflammatory process of the pancreas, with variable involvement of local or remote organ systems. The annual incidence of acute pancreatitis in the UK is approximately 30 per 100,000 of the population (1). This equates to approximately 18,000 people developing acute pancreatitis every year in the UK (1). There has been an increase in the incidence of pancreatitis in the last two decade (2). Gallstones and excessive alcohol are the two main causes for acute pancreatitis in the UK (3). Increasing age, male gender, and lower socioeconomic class are associated with a higher incidence of acute pancreatitis (1).

The clinical manifestation of acute pancreatitis is believed to be caused by activation of inflammatory pathways either directly by the pathologic insult or indirectly by activation of trypsin, a protease which can break down the pancreas (4). The average 60 day mortality associated with acute pancreatitis is 6.4% (1). Deaths occur as a result of a massive fluid extravasation from the inflamed pancreas combined with a severe systemic inflammatory response in the early stage and mainly from local complications in the late stage (5). The systemic complications occurring in the early phase result in poor oxygen delivery to the tissues and include worsening of pre-existing illnesses such as heart or chronic lung disease. Local complications which occur in the later phase include pancreatic pseudocysts, infected collections and pancreatic necrosis. These early and late complications are the major causes of the mortality associated with acute pancreatitis (6,7). They are also largely responsible for the decreased health-related quality of life and loss of work days following acute pancreatitis (8). Thus, acute pancreatitis has a major effect on UK society with its complications having a significant impact on patients, their next of kin, and their employers.

Various pharmacological interventions have been evaluated in acute pancreatitis, but none are in current clinical practice (9). Supportive management in terms of maintenance of fluid and electrolyte balance remains the mainstay in the treatment of acute pancreatitis. Despite

For peer review only

the key importance of fluid therapy, a recent systematic review has highlighted that there is a lack of information on the optimal fluid therapy in acute pancreatitis (9,10). The clinical presentation of acute pancreatitis varies from mild to severe life-threatening conditions. There is some evidence for aggressive fluid therapy with Hartmann's solution in those with severe disease. However, the rate and volume of fluid therapy is still debated for those with mild or moderate disease. Hence, high-quality randomised controlled trials (RCTs) in fluid therapy for acute pancreatitis are necessary.

Goal-directed fluid therapy (GDFT) is a complex intervention in which intravenous (IV) fluid is given to optimise haemodynamic variables. This is achieved by stroke volume optimisation using a cardiac output monitor. RCTs have shown that GDFT decreases complications and mortality in other situations associated with a systemic inflammatory response (11,12). Most of these trials however involved cardiac output monitoring for GDFT during surgery or in the intensive therapy unit (ITU) and not in a ward-based setting. For GDFT to be beneficial in acute pancreatitis, which involves a cascade of inflammatory events, therapy is likely to be most beneficial if commenced at the earliest opportunity to intervene following the onset of pancreatitis which would equate with the time of admission to hospital. There has been one randomised controlled trial on ward based GDFT in patients with acute pancreatitis comparing the effect of GDFT with Lactated ringers to normal saline on inflammatory response (13). However, the trial was based on optimisation of blood urea nitrogen (BUN) and not cardiac output parameters and failed to show a reduction in the inflammatory response or improved clinical outcomes. Whilst other endpoints such as heart rate (HR), urine output (UO), haematocrit levels and central venous pressures have been suggested for fluid therapy in acute pancreatitis, it is the optimisation of intravascular volume with fluid therapy guided by cardiac output measures that has been previously shown to be effective in decreasing complications and mortality in major surgery (14,15). There is currently no RCT investigating the role of ward based GDFT using cardiac output parameters as target in patients with acute pancreatitis.

For peer review only

With the development of non-invasive cardiac output monitors such as Cheetah NICOM™ (Cheetah Medical Ltd, Maidenhead, Berkshire, UK), it is possible to measure the cardiac output in a ward setting (16). Ward-based GDFT has the potential to decrease the early inflammatory response and hence complications related to acute pancreatitis which might result in decreased mortality and improved health-related quality of life (HRQoL). Reduced inflammation would also lead to a reduced intensive care unit (ICU) and hospital stay providing significant cost savings to the NHS, employers and care providers. This two-centre RCT will aim to assess the feasibility of guiding the initial 48 hours of IV fluid administration in patients with acute pancreatitis using ward-based GDFT.

Methods

Study design and setting

The GAP trial has been designed in accordance with the SPIRIT guidelines as a two centre, feasibility RCT (17). The study will primarily investigate the ability to recruit patients at the selected sites to a feasibility RCT of ward-based GDFT versus standard fluid therapy in patients with acute pancreatitis, the rate of withdrawal from GDFT protocol, and the reasons for withdrawal from GDFT protocol. We will also assess the safety and practicality of ward-based GDFT and collect outcome measures which can be evaluated as end points for a subsequent multi-centre study on efficacy. Indicative costs will be collected to inform a subsequent cost effectiveness study. The trial outline is illustrated in Figure 1 and the schedule of enrolment, interventions and assessments is shown in Table 1.

Participants

Adults (>16 years) admitted as an emergency with acute pancreatitis will be included. Acute pancreatitis must be confirmed by the international consensus criteria (table 2) (18). The exclusion criteria will be patients transferred for the management of complications of acute

For peer review only - http://bmjopen.bmj.com/site/about/guidelines.xhtml

pancreatitis, requiring immediate admission to the ICU, chronic pancreatitis in whom an acute exacerbation cannot be confirmed, a history of cardiac failure in the past three months and those unable to provide fully informed consent.

Recruitment

Patients referred from the emergency department for admission through the emergency surgical team will be screened by the general surgical registrar on-call. Due to the time-dependant nature of this trial, similar to most trauma and emergency surgery studies, a summarised and abbreviated patient information sheet (PIS) will be provided for patients initially on arrival and they will be subsequently provided with an in-depth version. Informed consent will be obtained by a member of the clinical or research team trained in Good Clinical Practice (GCP) (19). The trial nursing staff responsible for instituting the trial intervention will be contacted within four hours of diagnosis. Intervention or standard of care will commence within six hours of the diagnosis for the next 48 hours of inpatient stay. Since the primary goal of this study is to assess the feasibility of delivering *ward-based* GDFT, patients requiring escalation of care and admission to ICU during the intervention will only have continued cardiac output monitoring until completion of the 48 hour period.

Randomisation

This will be performed using ‘Sealed Envelope’, an internet-based randomisation system (www.sealedenvolpe.com). Eligible patients who have consented to take part in the trial will be 1:1 randomised to ward based GDFT or standard care, stratified by site on admission, prior to ward transfer. The trial research nurse responsible (unblinded) will log into the database and create randomisation codes for both groups online. Information on randomisation will be stored in the case report form (CRF).

Intervention

For peer review only - http://bmjopen.bmj.com/site/about/guidelines.xhtml

GDFT will be carried out for 48 hours. It can take up to 48 hours for the severity of pancreatitis to manifest and is considered the 'golden' period for interventions that may decrease severity (20). GDFT will be based on a standard algorithm which uses the stroke volume (SV) derived from non-invasive cardiac output monitoring using the Cheetah NICOM™ (figure 2). The fluid administration regimen will be as follows: maintenance fluid should be administered at 1.5 ml/kg/hr using a balanced crystalloid solution. On admission SV is recorded and an initial bolus of 250ml of IV fluid (balanced electrolyte solution) is given over 5 to 10 minutes. If there is a sustained rise in SV of greater than 10% for 15 minutes or more, this indicates fluid responsiveness and a repeat 250ml bolus will be given. If there is not a rise in SV of greater than 10% then the patient is deemed fluid unresponsive and no further fluid boluses are administered. SV monitoring continues four hourly and if it decreases by more than 10% a further fluid bolus is administered as above.

Control (standard care group)

The choice of fluid type, volume and rate of administration on the first 48 hours of admission for patients randomised to the control arm will be decided by the clinical team caring for the patient. Cardiac output monitoring variables will be measured every 4 hours on the standard care arm during the first 48 hours, however, the results will be blinded to the clinical team. The choice of fluid type, volume and rate will be recorded in detail. All other clinical, biochemical and HRQoL outcome measures will also be recorded for standard of care patients as outlined below in 'outcome measures' section.

The Cheetah NICOM™ device (Cheetah Medical Ltd, Maidenhead, Berkshire, UK) is a CE marked device has been purchased for this study and training has been arranged for all staff on the equipment and the associated fluid administration algorithm which will be a key component of the trial. The research team will follow hospital policy on signals for ICU outreach reviews and escalation of care to ICU. NICOM monitoring and outcome measures will be recorded in ICU for both control and GDFT arms.

For peer review only

Blinding

It will not be possible to blind the research or clinical team during the first 48 hours of the study. However, the participants, outcome assessors of health-related quality of life, and statisticians will be blinded to the groups. Patient blinding will be aided by NICOM monitoring of both intervention and control groups but performing GDFT in the intervention group alone. NICOM data from the control group will be collected at the same time points as the intervention group and will not be available to the treating clinicians.

Outcome measures

The primary outcome of the trial will be an assessment of feasibility. In particular, the ability to identify and recruit patients at the selected sites to a study of acute pancreatitis. A recruitment rate of at least 30% over 17-month trial period will be deemed as successful. We will also assess the availability of study team for a condition presenting as an emergency, ability to randomise and commence ward GDFT within 6 hours of admission, completion rate of 48 hours of GDFT, rate of withdrawal (less than 20%) from GDFT protocol, reasons for withdrawal from GDFT protocol and proportion of complications in the two groups.

Several secondary clinical, surgical, biochemical, safety and quality of life outcomes will also be collected. Mortality will be assessed as well. Clinical outcomes will include severity of pancreatitis as assessed by Glasgow score, proportion of patients with severe acute pancreatitis, necrotising pancreatitis, infected pancreatic necrosis, requiring ITU stay, requiring renal replacement therapy, requiring ventilation, requiring surgical interventions for complications related to pancreatitis and incidence of positive blood cultures. Resource use data for health economic analysis on length of hospital stay, length of ICU stay, and number of days ventilated, time to return to pre-pancreatitis activities, number of work days lost (in those who work), and costs (NHS and personal social services (PSS) perspectives) will be collected. Health-related quality of life (HRQoL) will be assessed using the well validated

For peer review only

EQ5D questionnaire on admission as baseline and subsequently on day 7, day 30 and day 90. All complications of pancreatitis including treatment-related adverse events (post-radiological and surgical complications as classified by Clavien-Dindo classification) and serious adverse events related to intervention will be recorded up to discharge and at follow-up. Routine biochemical tests performed as part of clinical care including blood gases, liver function tests, clotting profile, renal function, amylase, full blood count and C-reactive protein (CRP) will be recorded on admission and daily up to two weeks and bi-weekly up to discharge. In addition, serum samples will be collected from participants in both groups on admission, 12, 24- and 48-hour time points and stored at -80 degrees for future analysis of biomarkers in acute pancreatitis.

Follow-up

Participants will be followed for 90 days. All clinical and HRQoL outcomes will be measured up to discharge and specifically at 30 days and 90 days by face-to-face or telephone follow-up. CRP will be measured at 24 hours and 48 hours and every 24 hours during the hospital stay as per routine clinical practice.

Sample size calculation

Formal sample size calculations are not appropriate for a feasibility study, hence a convenient sample size of 50 patients, randomised equally to the two groups, has been chosen. Sample sizes between 24 and 50 have been recommended to estimate the standard deviation required for a sample size calculation for a subsequent large RCT aimed at evaluating the cost-effectiveness of GDFT (21).

Trial management structure

A Trial Management Group (TMG) chaired by the Chief Investigator and comprising of leads at different sites, ICU research nurses, the surgical team representative, research fellows, statistician, qualitative researchers and health economist will meet fortnightly to discuss trial

For peer review only

progress and address issues in recruitment and delivering the intervention. Trial progress will be reported to independent Data Monitoring Committee (DMC) comprising of experts in acute pancreatitis, medical statistics and clinical trials every 6 months.

All serious complications and any mortalities will be immediately referred to the sponsor and reported to the trial management group and independent data monitoring committee. The trial will be stopped in case of two or more treatment related suspected unexpected serious adverse reactions (SUSARs),

Data collection and analysis

Recruitment data which includes number of eligible patients presenting to the emergency department, number of patients screened, number of patients consenting, and number of patients randomised is recorded in a recruitment log in the emergency department. A CONSORT diagram will be presented to provide a detailed description of participant numbers at each time point during the trial (22). A paper CRF (Case Report Form) is created for each participant enrolled into the study. The data collected on the paper CRF is then transferred to a secure online database using the RedCap (Research Electronic Data Capture) platform.

Since this is a feasibility study, all analyses other than recruitment rate and withdrawal rates should be considered exploratory. The two groups will be compared to ensure they have similar baseline characteristics (table 3) using means and standard deviations or medians and inter-quartile ranges for continuous variables, as appropriate, and frequency counts and percentages for categorical variables.

For the primary outcome, i.e., feasibility, the proportion of patients who consent to be randomised and the rate of withdrawal from GDFT protocol will be presented with a 95% confidence interval. The median number of complications, graded by the Clavien-Dindo classification, in each group will be presented. The proportions of people with complications

For peer review only

between the two groups will be presented and compared using appropriate statistical test and 95% confidence interval.

For the secondary outcomes, amongst patients who participate in the trial, all clinical and surgical outcome measures will be presented for each group separately using the mean, standard deviation, median, minimum and maximum for continuous outcomes and using frequencies and proportions for categorical variables.

Quality of life will also be summarised for each group using mean profile plots over time. The mean difference in quality of life scores between the two groups at 7 days up will be presented with a 95% confidence interval.

Kaplan-Meier survival estimates and plots will be presented to compare survival rates between the two groups.

All other secondary outcomes collected over time will be summarised for each group using mean profile plots. Mean differences for continuous outcomes and difference in proportion for binary outcomes shall be presented, with appropriate 95% confidence intervals, at 30 and 90 days.

The number and nature of adverse events shall be reported for each group. No formal comparisons between the groups will be made and no hypothesis tests will be carried out. The results will inform us how sensitive the outcome measures are and, along with other information, will be used to determine the primary outcome of a subsequent large RCT. The results will also inform a sample size calculation for the primary outcome chosen.

Assessment of feasibility

For peer review only

This is a feasibility study and one aspect of this feasibility evaluation is the ability to recruit patients with acute pancreatitis into a trial of this nature. According to hospital episode statistics, an in-house review of admissions for acute pancreatitis to the Royal Free Hospital over the last three years showed 107 cases per year. After excluding patients who were transferred from another hospital for tertiary care, those who required immediate ICU admission, those with chronic pancreatitis in whom an acute exacerbation could not be confirmed by internal consensus criteria, current or past cardiac failure, or unable to provide fully informed consent, 80 patients per year would be eligible for this trial from a single centre. With a second centre we would anticipate 120 suitable patients per year. We anticipate recruiting at least 30% of potential participants which equates to 36 participants annually and a recruitment time of 17 months to recruit the 50 patients.

For feasibility assessment, a recruitment log will be used to collect information on suitability and consent rate as well as availability of trials team for GDFT, consent, failure to commence ward GDFT, withdrawal with reasons and failure to complete the study. In case of lower than anticipated recruitment at six months, we will perform the following; a) analyse the results of the qualitative research earlier and identify the reasons for poor recruitment (and take actions to resolve the problems) if we get the anticipated number of patients screened but the recruitment rate is lower than anticipated, b) recruitment of additional centres if we do not get the anticipated number of potential participants screened but the recruitment rate is at least as good as the anticipated rate c) a combination of the above if the number of potential participants screened and the recruitment rate are lower than anticipated.

We will explore the reasons for participation and non-participation of eligible patients and patients' and clinicians' acceptability of the trial to assist in optimisation of recruitment strategies employed for the definitive trial. Non-participation can be related to how the clinical trial is presented to the patient, and how the patient assimilates this information. It is therefore important to understand how patients perceive information about potential participation and

For peer review only

their experiences of receiving information relating to the trial. Interviews with a sample of eligible patients will explore patient perspectives of treatment, their understanding of the two treatments, reasons for taking part or refusing the trial, and the acceptability of randomisation between the procedures. Interviews with clinical staff will explore their views about the trial, clinical equipoise, and their understanding of the recruitment challenges. Semi-structured interviews will be informed by a topic guide developed in conjunction with the trial management group which includes patient representatives. Patient information sessions (recruiter meetings) will be audio recorded to examine how information is presented, and identify issues potentially affecting trial recruitment. Patient information sessions will be analysed at an early stage if there is poor recruitment and will inform the development of any additional training materials for recruitment for the definitive trial.

Progress to full trial

The criteria to progress to a subsequent full trial will be determined quantitatively as a) consent rate of at least 30%, the ability to recruit 50 patients to the study at the two sites over 17 months, b) GDFT can be successfully performed within 6 hours of diagnosis of acute pancreatitis and can be continued until at least 48 hours after admission in a minimum of 80% of participants randomised to GDFT, and c) the complication rate in the intervention group is not more than 10% higher than that of the control group at 90 days. A cut off of 30% has been chosen for recruitment as this would be an achievable target in the definitive trial. A lower recruitment rate may indicate a lack of acceptability among clinicians and/or patients to participate in the definitive trial. In addition, a recruitment rate less than 30% is likely to make the subsequent definitive trial very expensive. A less than 30% recruitment may also highlight issues related to generalisability of the results, indicating a reduction in value of the subsequent definitive trial in terms of applicability in NHS.

Dissemination

For peer review only

The results of this study will be presented to the national and international meetings with interest in the management of acute pancreatitis and prepared for publication in peer-reviewed journals with a readership in general surgery and critical care.

Discussion

Acute pancreatitis is an inflammatory disease associated with a high mortality rate (23). Adequate fluid resuscitation has been identified as a key intervention in prevention of systemic complications, however, the debate over the type, rate, or amount of intravenous fluids to be administered is ongoing (24). With the advent of non-invasive cardiac output monitoring (NICOM) devices, delivering haemodynamic guided fluid replacement is now possible in patients admitted to the general ward. The GAP trial is the first ward-based RCT of GDFT in acute pancreatitis. The trial will assess feasibility and safety of delivering this intervention in the ward and if successful, will progress to a multicentre trial to assess efficacy and cost-effectiveness.

Trial Status

The GAP trial opened for recruitment on 8th January 2018 for a period of 18 months.

List of Abbreviations

- C-Reactive Protein (CRP)
- Data Monitoring Committee (DMC)
- GDFT in Acute Pancreatitis (GAP)
- Goal Directed Fluid Therapy (GDFT)
- Health-Related Quality of Life (HRQoL)
- Intensive Care Unit (ICU)
- Intravenous (IV)
- National Health System (NHS)
- Non-Invasive Cardiac Output Monitor (NICOM)

For peer review only

- Patient Information Sheet (PIS)
- Personal Social Services (PSS)
- Randomized Control Trial (RCT)
- Stroke Volume (SV)
- Suspected Unexpected Serious Adverse Reactions (SUSAR)
- Trial Management Group (TMG)

Declarations

Ethical approval and consent to participate

The trial protocol (v2) has been reviewed and approved by the London Central Research Ethics Committee (REC ref: 17/LO/1235, project ID: 221872). Informed consent will be obtained from eligible patients after screening by a member of the research or clinical team trained in Good Clinical Practice (GCP). The trial has been registered on ISRCTN (ISRCTN 36077283) on 09 April 2018 (http://www.isrctn.com/ISRCTN36077283).

Consent for publication

Not applicable.

Availability of data and materials

Data from this trial will be made available after analysis and publication upon reasonable request from the corresponding author.

Competing interests

The authors declare that they have no competing interests.

Funding

For peer review only

This study is funded by the National Institute for Health Research (NIHR) Research for Patient Benefit Programme (RfPB) (Grant Reference Number PB-PG-0815-20002). This paper presents independent research funded by the (NIHR) under its Research for Patient Benefit (RfPB) Programme (Grant Reference Number PB-PG-0815-20002). The views expressed are those of the author(s) and not necessarily those of the NHS, the NIHR or the Department of Health.

Author contributions

FF manuscript preparation, mechanistic components of protocol design, trial and database management, FS manuscript preparation, trial management, FR statistical analysis plan, KG major contribution to design and funding application, DM protocol design of GDFT regimes, trial management, SS initial protocol developments and ethical approvals, CE, MC, OS, GA and HF trial research nurses trained in delivering GDFT, data collection, JS health economic analysis design, MV participant enrolment planning through emergency surgery , BD principal investigator and grant holder, overall trial management, study design and review of manuscript. All authors read and approved the final manuscript.

Acknowledgements

We are grateful for the support of our emergency surgical colleagues Mr Olegunju Ogunbiyi, Mr Jonathan Knowles, Mr Rovan D'Souza, Mr Colin Hart, Mr Ahmad Hamad, the surgical registrars Ms Tanzeela Gala, Ms Karen Bosch, Ms Aliza Abeles, Mr Guillaume Lafaurie, Mr Tom Hanna, Mr Stephanos Pericleous, our emergency department lead Dr Jonathan Costello, without whom the conduct of this trial would not be possible. We gratefully acknowledge the financial support of the Wellington hospital, London, in their HPB Surgery Fellowship funding for Mr Farid Froghi.

References

1. Roberts SE, Akbari A, Thorne K, Atkinson M, Evans PA. The incidence of acute

For peer review only - http://bmjopen.bmj.com/site/about/guidelines.xhtml

459 pancreatitis: Impact of social deprivation, alcohol consumption, seasonal and demographic factors. Aliment Pharmacol Ther [Internet]. Wiley-Blackwell; 2013 Sep [cited 2018 Sep 5];38(5):539–48. Available from: http://www.ncbi.nlm.nih.gov/pubmed/23859492

463 2. Working Party of the British Society of Gastroenterology, Association of Surgeons of Great Britain and Ireland, Pancreatic Society of Great Britain and Ireland, Association of Upper GI Surgeons of Great Britain and Ireland. UK guidelines for the management of acute pancreatitis. Gut [Internet]. 2005;54(suppl 3):iii1-iii9. Available from: http://gut.bmj.com/content/54/suppl_3/iii1

468 3. Párniczky A, Kui B, Szentesi A, Balázs A, Szûcs Á, Mosztbacher D, et al. Prospective, Multicentre, Nationwide Clinical Data from 600 Cases of Acute Pancreatitis. PLoS One. 2016;11(10):1–19.

471 4. Leung PS, Ip SP. Pancreatic acinar cell: Its role in acute pancreatitis. Vol. 38, International Journal of Biochemistry and Cell Biology. 2006. p. 1024–30.

473 5. Andersson R, Andersson B, Andersson E, Axelsson J, Eckerwall G, Tingstedt B. Acute pancreatitis - From cellular signalling to complicated clinical course. Hpb. 2007;9(6):414–20.

476 6. 'tHart NA, Leuvenink HGD, Ploeg RJ. New solutions in organ preservation. Transplant Rev [Internet]. W.B. Saunders; 2002 Jul 1 [cited 2017 Dec 11];16(3):131–41. Available from: http://www.sciencedirect.com/science/article/pii/S0955470X02800027

479 7. De Beaux AC, Palmer KR, Carter DC. Factors influencing morbidity and mortality in acute pancreatitis; an analysis of 279 cases. Gut [Internet]. BMJ Publishing Group; 1995 Jul [cited 2018 Sep 14];37(1):121–6. Available from: http://www.ncbi.nlm.nih.gov/pubmed/7672660

483 8. Vege SS, Gardner TB, Chari ST, Munukuti P, Pearson RK, Clain JE, et al. Low Mortality and High Morbidity in Severe Acute Pancreatitis Without Organ Failure: A Case for Revising the Atlanta Classification to Include "Moderately Severe Acute Pancreatitis." Am J Gastroenterol [Internet]. Nature Publishing Group; 2009 Mar 3

For peer review only

[cited 2018 Sep 14];104(3):710–5. Available from: http://www.nature.com/articles/ajg200877

9. Moggia E, Koti R, Belgaumkar AP, Fazio F, Pereira SP, Davidson BR, et al. Pharmacological interventions for acute pancreatitis. Vol. 2017, Cochrane Database of Systematic Reviews. 2017. p. 1–14.

10. Iqbal U, Anwar H, Scribani M. Ringer's Lactate vs Normal Saline in Acute Pancreatitis: A Systematic Review and meta- analysis.

11. Pearse RM, Harrison DA, MacDonald N, Gillies MA, Blunt M, Ackland G, et al. Effect of a Perioperative, Cardiac Output–Guided Hemodynamic Therapy Algorithm on Outcomes Following Major Gastrointestinal Surgery. JAMA [Internet]. American Medical Association; 2014 Jun 4 [cited 2018 Oct 11];311(21):2181. Available from: http://jama.jamanetwork.com/article.aspx?doi=10.1001/jama.2014.5305

12. Hamilton MA, Cecconi M, Rhodes A. A Systematic Review and Meta-Analysis on the Use of Preemptive Hemodynamic Intervention to Improve Postoperative Outcomes in Moderate and High-Risk Surgical Patients. Anesth Analg [Internet]. 2011 Jun [cited 2016 Aug 27];112(6):1392–402. Available from: http://content.wkhealth.com/linkback/openurl?sid=WKPTLP:landingpage&an=00000539-201106000-00027

13. Wu BU, Hwang JQ, Gardner TH, Repas K, Delee R, Yu S, et al. Lactated Ringer's Solution Reduces Systemic Inflammation Compared With Saline in Patients With Acute Pancreatitis. Clin Gastroenterol Hepatol. 2011;9(8):710–7.

14. Rivers EP, Ander DS, Powell D. Central venous oxygen saturation monitoring in the critically ill patient. Curr Opin Crit Care. 2001;7(3):204–11.

15. Bortolotti P, Saulnier F, Colling D, Redheuil A, Preau S. New tools for optimizing fluid resuscitation in acute pancreatitis. World J Gastroenterol [Internet]. Baishideng Publishing Group Inc; 2014 Nov 21 [cited 2018 Oct 11];20(43):16113–22. Available from: http://www.ncbi.nlm.nih.gov/pubmed/25473163

16. Dunham CM, Chirichella TJ, Gruber BS, Ferrari JP, Martin JA, Luchs BA, et al.

For peer review only

Emergency department noninvasive (NICOM) cardiac outputs are associated with trauma activation, patient injury severity and host conditions and mortality. J Trauma Acute Care Surg. 2012;73(2):479–85.

17. Chan A-W, Tetzlaff JM, Gøtzsche PC, Altman DG, Mann H, Berlin JA, et al. SPIRIT 2013 explanation and elaboration: guidance for protocols of clinical trials. BMJ [Internet]. BMJ Group; 2013 Jan 8 [cited 2016 Oct 25];346:e7586. Available from: http://www.ncbi.nlm.nih.gov/pubmed/23303884

18. Tenner S, Baillie J, Dewitt J, Vege SS. American college of gastroenterology guideline: Management of acute pancreatitis. Am J Gastroenterol. Nature Publishing Group; 2013;108(9):1400–15.

19. Good Clinical Practice (GCP) [Internet]. [cited 2018 Sep 14]. Available from: https://www.nihr.ac.uk/our-faculty/clinical-research-staff/learning-and-development/national-directory/good-clinical-practice/

20. Fisher JM, Gardner TB. The “Golden Hours” of Management in Acute Pancreatitis. Am J Gastroenterol [Internet]. Nature Publishing Group; 2012 Aug 3 [cited 2018 Oct 11];107(8):1146–50. Available from: http://www.nature.com/articles/ajg201291

21. Sim J, Lewis M. The size of a pilot study for a clinical trial should be calculated in relation to considerations of precision and efficiency. J Clin Epidemiol [Internet]. Elsevier; 2012 Mar 1 [cited 2018 Sep 15];65(3):301–8. Available from: http://www.ncbi.nlm.nih.gov/pubmed/22169081

22. Moher D, Schulz KF, Altman DG. The CONSORT statement: revised recommendations for improving the quality of reports of parallel-group randomised trials. Lancet [Internet]. Elsevier; 2001 Apr 14 [cited 2018 Dec 22];357(9263):1191–4. Available from: https://www.sciencedirect.com/science/article/pii/S0140673600043373?via%3Dihub

23. Bank S, Singh P, Pooran N, Stark B. Evaluation of Factors That Have Reduced Mortality From Acute Pancreatitis Over the Past 20 Years. J Clin Gastroenterol [Internet]. 2002;35(1). Available from:

For peer review only

https://journals.lww.com/jcge/Fulltext/2002/07000/Evaluation_of_Factors_That_Have_Reduced_Mortality.12.aspx

24. Aggarwal A, Manrai M, Kochhar R. Fluid resuscitation in acute pancreatitis. World J Gastroenterol [Internet]. Baishideng Publishing Group Inc; 2014 Dec 28 [cited 2018 Sep 15];20(48):18092–103. Available from: http://www.ncbi.nlm.nih.gov/pubmed/25561779

For peer review only

Table 1. Schedule of enrolment, interventions and assessments

Procedures	Enrolment	Allocation	Post-allocation								Close-out
**Time point**	**-t0**	**0**	**0 hrs**	**12 hrs**	**24 hrs**	**48 hrs**	**discharge**	**7 days**	**30 days**	**90 days**	
**ENROLMENT**											
Eligibility screen	x										
PIS given	x										
Informed consent	x										
Registration	x										
Allocation		x									
**INTERVENTION**											
GDFT (intervention)			◆	—	—	◆					
Standard care (control)			◆	—	—	◆					
ASSESSMENTS											
NICOM Monitoring			◆	—	—	◆					
Blood tests			x	x	x	x					
HRQoL			x					x	x	x	x
Complications			x	x	x	x	x		x	x	x
Other outcomes			x	x	x	x	x		x	x	x

For peer review only

Table 2. International consensus criteria for acute pancreatitis

Acute pancreatitis diagnosis confirmed with two of the following three features:
• Abdominal pain consistent with acute pancreatitis (acute onset of a persistent, severe, epigastric pain often radiating to the back)<br>• Serum amylase or lipase activity at least three times greater than the upper limit of normal.<br>• Characteristic findings of acute pancreatitis on contrast-enhanced computed tomography (CECT) and less commonly magnetic resonance imaging (MRI) or transabdominal ultrasonography.

For peer review only

Table 3. Baseline characteristics of patients

Age
Sex
Ethnicity
BMI
Time since onset of symptoms
Presumed cause of pancreatitis
Comorbidities
Baseline observations
Baseline renal function
Baseline blood gas parameters
Glasgow severity score on admission
Intravenous fluids prior to intervention

For peer review only

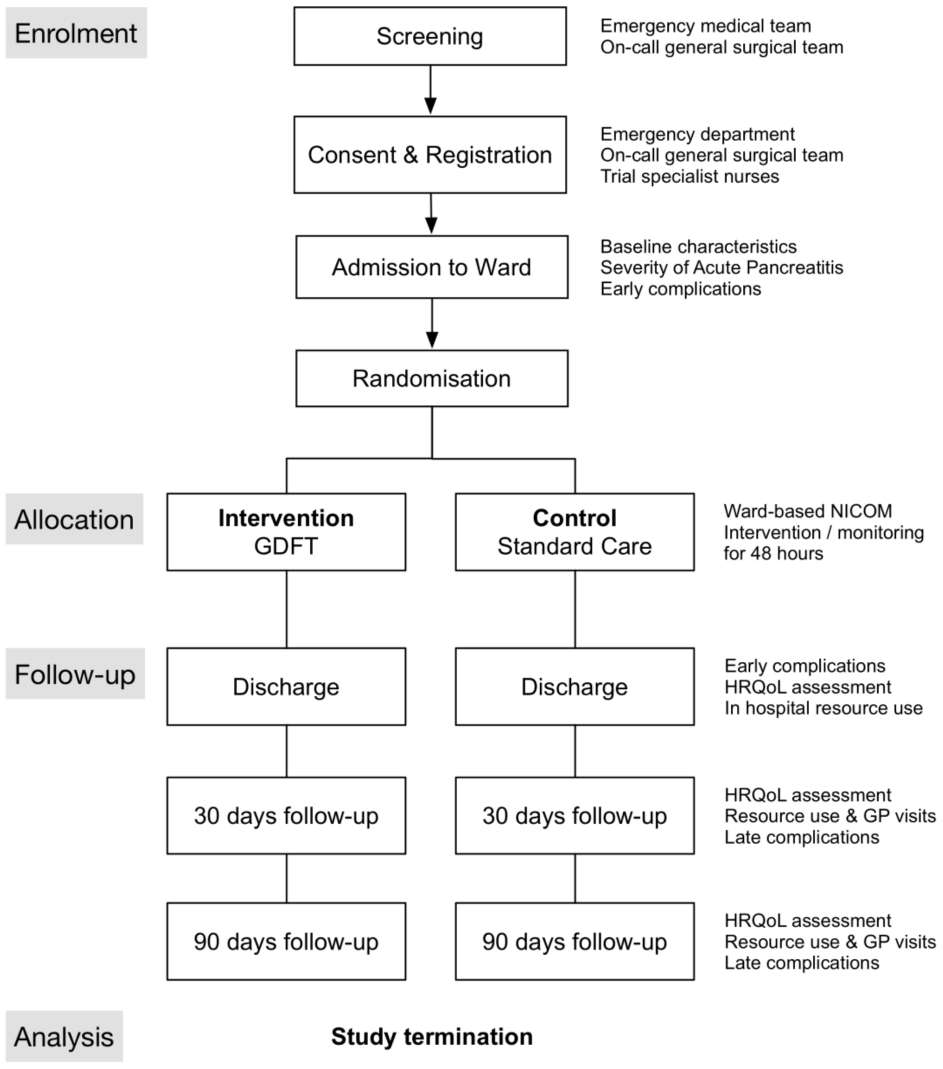

Figure 1 Trial flow diagram

207x234mm (300 x 300 DPI)

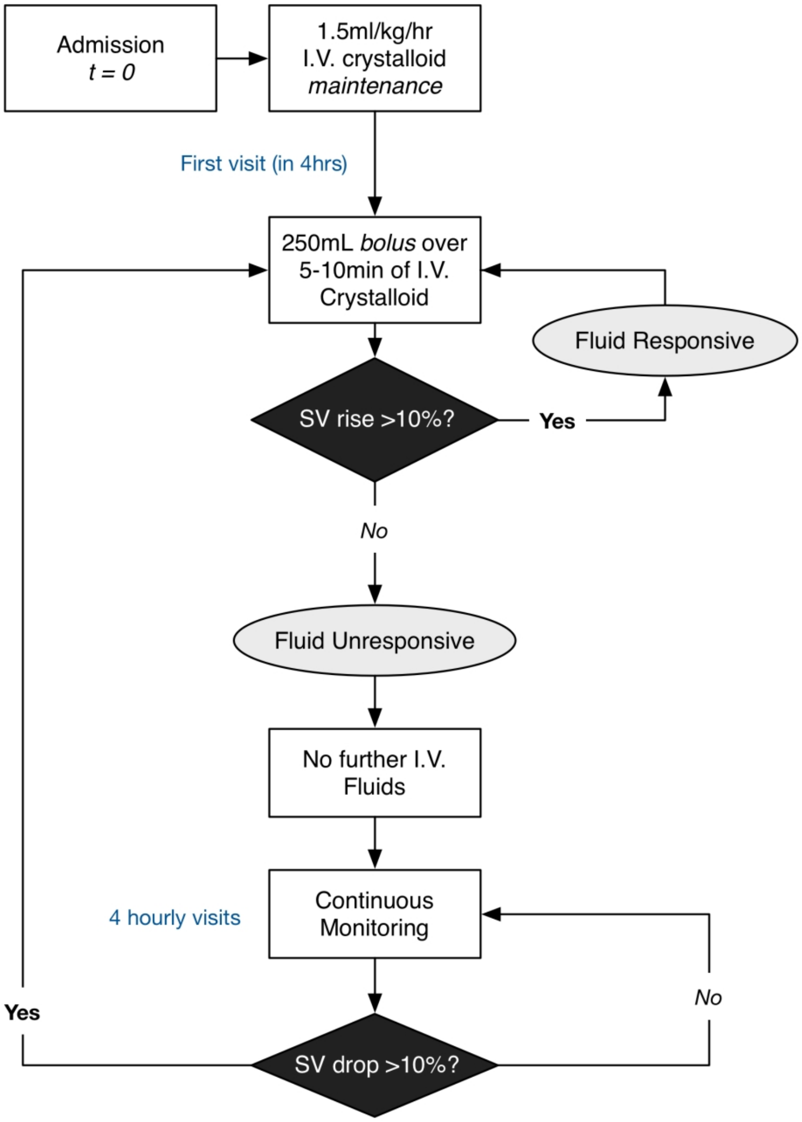

Figure 2 Goal-directed fluid therapy protocol

155x216mm (300 x 300 DPI)

Reporting checklist for protocol of a clinical trial.

Based on the SPIRIT guidelines.

Instructions to authors

Complete this checklist by entering the page numbers from your manuscript where readers will find each of the items listed below.

Your article may not currently address all the items on the checklist. Please modify your text to include the missing information. If you are certain that an item does not apply, please write "n/a" and provide a short explanation.

Upload your completed checklist as an extra file when you submit to a journal.

In your methods section, say that you used the SPIRIT reporting guidelines, and cite them as:

Chan A-W, Tetzlaff JM, Altman DG, Laupacis A, Gøtzsche PC, Krleža-Jerić K, Hróbjartsson A, Mann H, Dickersin K, Berlin J, Doré C, Parulekar W, Summerskill W, Groves T, Schulz K, Sox H, Rockhold FW, Rennie D, Moher D. SPIRIT 2013 Statement: Defining standard protocol items for clinical trials. Ann Intern Med. 2013;158(3):200-207

		Reporting Item	Page Number
Title	#1	Descriptive title identifying the study design, population, interventions, and, if applicable, trial acronym	1
Trial registration	#2a	Trial identifier and registry name. If not yet registered, name of intended registry	2
Trial registration: data set	#2b	All items from the World Health Organization Trial Registration Data Set	2
Protocol version	#3	Date and version identifier	16
Funding	#4	Sources and types of financial, material, and other support	17
Roles and responsibilities: contributorship	#5a	Names, affiliations, and roles of protocol contributors	17
Roles and responsibilities:	#5b	Name and contact information for the trial sponsor	17

For peer review only

sponsor contact information			
Roles and responsibilities: sponsor and funder	#5c	Role of study sponsor and funders, if any, in study design; collection, management, analysis, and interpretation of data; writing of the report; and the decision to submit the report for publication, including whether they will have ultimate authority over any of these activities	17
Roles and responsibilities: committees	#5d	Composition, roles, and responsibilities of the coordinating centre, steering committee, endpoint adjudication committee, data management team, and other individuals or groups overseeing the trial, if applicable (see Item 21a for data monitoring committee)	17, 10
Background and rationale	#6a	Description of research question and justification for undertaking the trial, including summary of relevant studies (published and unpublished) examining benefits and harms for each intervention	4-6
Background and rationale: choice of comparators	#6b	Explanation for choice of comparators	4-6 / 8
Objectives	#7	Specific objectives or hypotheses	6
Trial design	#8	Description of trial design including type of trial (eg, parallel group, crossover, factorial, single group), allocation ratio, and framework (eg, superiority, equivalence, non-inferiority, exploratory)	6
Study setting	#9	Description of study settings (eg, community clinic, academic hospital) and list of countries where data will be collected. Reference to where list of study sites can be obtained	6
Eligibility criteria	#10	Inclusion and exclusion criteria for participants. If applicable, eligibility criteria for study centres and individuals who will perform the interventions (eg, surgeons, psychotherapists)	6
Interventions: description	#11a	Interventions for each group with sufficient detail to allow replication, including how and when they will be administered	8
Interventions: modifications	#11b	Criteria for discontinuing or modifying allocated interventions for a given trial participant (eg, drug dose change in response to harms, participant request, or improving / worsening disease)	8
Interventions:	#11c	Strategies to improve adherence to intervention protocols, and any	8

For peer review only

adherance		procedures for monitoring adherence (eg, drug tablet return; laboratory tests)	
Interventions: concomitant care	#11d	Relevant concomitant care and interventions that are permitted or prohibited during the trial	8
Outcomes	#12	Primary, secondary, and other outcomes, including the specific measurement variable (eg, systolic blood pressure), analysis metric (eg, change from baseline, final value, time to event), method of aggregation (eg, median, proportion), and time point for each outcome. Explanation of the clinical relevance of chosen efficacy and harm outcomes is strongly recommended	9
Participant timeline	#13	Time schedule of enrolment, interventions (including any run-ins and washouts), assessments, and visits for participants. A schematic diagram is highly recommended (see Figure)	Table 1
Sample size	#14	Estimated number of participants needed to achieve study objectives and how it was determined, including clinical and statistical assumptions supporting any sample size calculations	10
Recruitment	#15	Strategies for achieving adequate participant enrolment to reach target sample size	7
Allocation: sequence generation	#16a	Method of generating the allocation sequence (eg, computer-generated random numbers), and list of any factors for stratification. To reduce predictability of a random sequence, details of any planned restriction (eg, blocking) should be provided in a separate document that is unavailable to those who enrol participants or assign interventions	7
Allocation concealment mechanism	#16b	Mechanism of implementing the allocation sequence (eg, central telephone; sequentially numbered, opaque, sealed envelopes), describing any steps to conceal the sequence until interventions are assigned	7
Allocation: implementation	#16c	Who will generate the allocation sequence, who will enrol participants, and who will assign participants to interventions	7
Blinding (masking)	#17a	Who will be blinded after assignment to interventions (eg, trial participants, care providers, outcome assessors, data analysts), and how	9
Blinding (masking):	#17b	If blinded, circumstances under which unblinding is permissible,	9

For peer review only

emergency unblinding		and procedure for revealing a participant's allocated intervention during the trial	
Data collection plan	#18a	Plans for assessment and collection of outcome, baseline, and other trial data, including any related processes to promote data quality (eg, duplicate measurements, training of assessors) and a description of study instruments (eg, questionnaires, laboratory tests) along with their reliability and validity, if known. Reference to where data collection forms can be found, if not in the protocol	11
Data collection plan: retention	#18b	Plans to promote participant retention and complete follow-up, including list of any outcome data to be collected for participants who discontinue or deviate from intervention protocols	11
Data management	#19	Plans for data entry, coding, security, and storage, including any related processes to promote data quality (eg, double data entry; range checks for data values). Reference to where details of data management procedures can be found, if not in the protocol	11
Statistics: outcomes	#20a	Statistical methods for analysing primary and secondary outcomes. Reference to where other details of the statistical analysis plan can be found, if not in the protocol	11-13
Statistics: additional analyses	#20b	Methods for any additional analyses (eg, subgroup and adjusted analyses)	11
Statistics: analysis population and missing data	#20c	Definition of analysis population relating to protocol non-adherence (eg, as randomised analysis), and any statistical methods to handle missing data (eg, multiple imputation)	11
Data monitoring: formal committee	#21a	Composition of data monitoring committee (DMC); summary of its role and reporting structure; statement of whether it is independent from the sponsor and competing interests; and reference to where further details about its charter can be found, if not in the protocol. Alternatively, an explanation of why a DMC is not needed	
Data monitoring: interim analysis	#21b	Description of any interim analyses and stopping guidelines, including who will have access to these interim results and make the final decision to terminate the trial	10
Harms	#22	Plans for collecting, assessing, reporting, and managing solicited and spontaneously reported adverse events and other unintended effects of trial interventions or trial conduct	11

For peer review only

Auditing	#23	Frequency and procedures for auditing trial conduct, if any, and whether the process will be independent from investigators and the sponsor	11
Research ethics approval	#24	Plans for seeking research ethics committee / institutional review board (REC / IRB) approval	16
Protocol amendments	#25	Plans for communicating important protocol modifications (eg, changes to eligibility criteria, outcomes, analyses) to relevant parties (eg, investigators, REC / IRBs, trial participants, trial registries, journals, regulators)	11
Consent or assent	#26a	Who will obtain informed consent or assent from potential trial participants or authorised surrogates, and how (see Item 32)	N/A
Consent or assent: ancillary studies	#26b	Additional consent provisions for collection and use of participant data and biological specimens in ancillary studies, if applicable	N/A
Confidentiality	#27	How personal information about potential and enrolled participants will be collected, shared, and maintained in order to protect confidentiality before, during, and after the trial	11
Declaration of interests	#28	Financial and other competing interests for principal investigators for the overall trial and each study site	16
Data access	#29	Statement of who will have access to the final trial dataset, and disclosure of contractual agreements that limit such access for investigators	16
Ancillary and post trial care	#30	Provisions, if any, for ancillary and post-trial care, and for compensation to those who suffer harm from trial participation	N/A
Dissemination policy: trial results	#31a	Plans for investigators and sponsor to communicate trial results to participants, healthcare professionals, the public, and other relevant groups (eg, via publication, reporting in results databases, or other data sharing arrangements), including any publication restrictions	15
Dissemination policy: authorship	#31b	Authorship eligibility guidelines and any intended use of professional writers	N/A
Dissemination policy: reproducible research	#31c	Plans, if any, for granting public access to the full protocol, participant-level dataset, and statistical code	15
Informed consent	#32	Model consent form and other related documentation given to	Appendix

For peer review only

materials		participants and authorised surrogates	
Biological specimens	#33	Plans for collection, laboratory evaluation, and storage of biological specimens for genetic or molecular analysis in the current trial and for future use in ancillary studies, if applicable	10

The SPIRIT checklist is distributed under the terms of the Creative Commons Attribution License CC-BY-ND 3.0. This checklist can be completed online using https://www.goodreports.org/, a tool made by the EQUATOR Network in collaboration with Penelope.ai

For peer review only

BMJ Open

Ward based Goal-Directed Fluid Therapy (GDFT) in Acute Pancreatitis (GAP trial): study protocol for a feasibility randomised controlled trial

Journal:	*BMJ Open*
Manuscript ID	bmjopen-2018-028783.R1
Article Type:	Protocol
Date Submitted by the Author:	02-Jul-2019
Complete List of Authors:	Froghi, Farid; University College London, Division of Surgery & Interventional Science; Royal Free Hospital, HPB and Liver Transplantation Surgery<br>Soggiu, Fiammetta; Royal Free Hospital, HPB and Liver Transplantation Surgery<br>Ricciardi, Federico; University College London, Statistical Sciences<br>Gurusamy, Kurinchi; University College London, Division of Surgery & Interventional Sciences<br>Martin, Daniel S.; University College London, Division of Surgery & Interventional Sciences; Royal Free Hospital, Critical Care Unit<br>Singh, Jeshika; PHMR Ltd, Health Economics<br>Siddique, Sulman; University College London, Division of Surgery & Interventional Sciences<br>Eastgate, Christine; Royal Free Hospital, Critical Care Unit<br>Ciaponi, Maria; Royal Free Hospital, Critical Care Unit<br>McNeil, Margaret; Royal Free Hospital, Critical Care Unit<br>Filipe, Helder; Royal Free Hospital, Critical Care Unit<br>Schwalowsky-Monks, Otto; Royal Free Hospital, Critical Care Unit<br>Asis, Gretchelle; Royal Free Hospital, Critical Care Unit<br>Varcada, Massimo; Royal Free Hospital, General and Emergency Surgery<br>Davidson, Brian; University College London, Division of Surgery & Interventional Sciences; Royal Free Hospital, HPB and Liver Transplantation Surgery
**Primary Subject Heading**:	Surgery
Secondary Subject Heading:	Gastroenterology and hepatology, Research methods
Keywords:	Acute Pancreatitis, Goal-Directed Fluid Therapy, Fluid Therapy, Cardiac Output

SCHOLARONE™
Manuscripts

Ward based Goal-Directed Fluid Therapy (GDFT) in Acute Pancreatitis (GAP trial): study protocol for a feasibility randomised controlled trial

Farid Froghi[1], Fiammetta Soggiu[1], Federico Ricciardi[5], Kurinchi Gurusamy[1], Daniel Martin[1,2], Jeshika Singh[4], Sulman Siddique[1], Christine Eastgate[2], Maria Ciaponi[2], Margaret McNeil[2], Helder Filipe[2], Otto Schwalowsky-Monks[2], Gretchelle Asis[2], Massimo Varcada[1,3], Brian Davidson[1]

[1]UCL Division of Surgery and Interventional Science, Royal Free Hospital, London

[2]Critical Care Unit, Royal Free Hospital, London

[3]General and Emergency Surgery Department, Royal Free Hospital, London

[4]PHMR, Health Economics, Pricing & Reimbursement, London, United Kingdom

[5]Department of Statistical Science, University College London, London

Corresponding author: Mr Farid Froghi
UCL Division of Surgery & Interventional Sciences
9th Floor, Royal Free Hospital
Pond Street
London NW3 2QG

Email: farid.froghi@nhs.net

Tel: 020 7794 0500

Abstract

Introduction: Acute pancreatitis is an inflammatory disease of the pancreas with high risk of developing multiorgan failure and death. There are no effective pharmacological interventions used in current clinical practice. Maintaining fluid and electrolyte balance is the mainstay of supportive management. Goal directed fluid therapy (GDFT) has been shown to decrease morbidity and mortality in surgical conditions with systemic inflammatory response. There is currently no randomized controlled trial (RCT) investigating the role of GDFT based on cardiac output parameters in patients with acute pancreatitis in the ward setting. A feasibility trial was designed to determine patient and clinician support for recruitment into a RCT of ward-based GDFT in acute pancreatitis, adherence to a GDFT protocol, safety, participant withdrawal, and to determine appropriate endpoints for a subsequent larger trial to evaluate efficacy.

Methods and Analysis: The GDFT in Acute Pancreatitis (GAP) trial is a prospective two-centre, feasibility RCT. Eligible adults admitted with new onset of acute pancreatitis will be enrolled and randomised into ward-based GDFT (n=25) or standard fluid therapy (n=25) within 6 hours from the diagnosis and continuing for the following 48 hours. Cardiac output parameters will be monitored with a non-invasive device (Cheetah NICOM ®; Cheetah Medical Ltd, Maidenhead, Berkshire, UK). The intervention will consist of a protocoled GDFT approach consisting of stroke volume optimisation with crystalloid fluid boluses, while the control group will receive standard care fluid therapy as advised by the clinical team. The primary endpoint is feasibility. Secondary endpoints will include safety of the intervention, complications, mortality, admission to intensive care unit, cost and quality of life.

Ethics and Dissemination: Ethics approved by the London Central Research Ethics Committee (17/LO/1235, project-ID: 221872). The results of this trial will be presented in international conference with interest in general surgery and acute care and published in a peer-reviewed journal.

Trial registration: ISRCTN 36077283 (26-03-2018)

For peer review only

Keywords:

Fluid therapy, Acute Pancreatitis, Cardiac Output, Goal-directed Fluid Therapy

Strengths and limitations of this study

- Adequate fluid therapy is a major determinant of outcomes in Acute Pancreatitis, however, there is no consensus on the rate, type and volume of fluids to be administered. Goal-directed fluid therapy (GDFT) based on stroke volume (SV) optimisation has been used after major surgery with a reduction in complications, however, delivering this intervention on the ward has been challenging without invasive monitoring.
- Novel development of non-invasive cardiac output monitoring (NICOM) technologies allows GDFT to be delivered in the ward based on haemodynamic parameters. This trial is the first GDFT randomised trial in acute pancreatitis using non-invasive cardiac output monitoring in the ward setting for SV optimisation.
- The golden period for intervention in acute pancreatitis is in the early phase of disease to prevent progression to severe inflammatory processes and complications. A major challenge in this trial is recruitment and delivering the intervention in the early hours of unplanned admission which is shared across trials in emergency care.

For peer review only

Introduction

Acute pancreatitis is a sudden onset inflammatory process of the pancreas, with variable involvement of local or remote organ systems. The annual incidence of acute pancreatitis in the UK is approximately 30 per 100,000 of the population (1). This equates to approximately 18,000 people developing acute pancreatitis every year in the UK (1). There has been an increase in the incidence of pancreatitis in the last two decade (2). Gallstones and excessive alcohol are the two main causes for acute pancreatitis in the UK (3). Increasing age, male gender, and lower socioeconomic class are associated with a higher incidence of acute pancreatitis (1).

The clinical manifestation of acute pancreatitis is believed to be caused by activation of inflammatory pathways either directly by the pathologic insult or indirectly by activation of trypsin, a protease which can break down the pancreas (4). The average 60 day mortality associated with acute pancreatitis is 6.4% (1). Deaths occur as a result of a massive fluid extravasation from the inflamed pancreas combined with a severe systemic inflammatory response in the early stage and mainly from local complications in the late stage (5). The systemic complications occurring in the early phase result in poor oxygen delivery to the tissues and include worsening of pre-existing illnesses such as heart or chronic lung disease. Local complications which occur in the later phase include pancreatic pseudocysts, infected collections and pancreatic necrosis. These early and late complications are the major causes of the mortality associated with acute pancreatitis (6,7). They are also largely responsible for the decreased health-related quality of life and loss of work days following acute pancreatitis (8). Thus, acute pancreatitis has a major effect on UK society with its complications having a significant impact on patients, their next of kin, and their employers.

Various pharmacological interventions have been evaluated in acute pancreatitis, but none are in current clinical practice (9). Supportive management in terms of maintenance of fluid and electrolyte balance remains the mainstay in the treatment of acute pancreatitis. Despite

For peer review only

the key importance of fluid therapy, a recent systematic review has highlighted that there is a lack of information on the optimal fluid therapy in acute pancreatitis (9,10). There has also been a decline in research in treatments for acute pancreatitis in general (11).

The clinical presentation of acute pancreatitis varies from mild to severe life-threatening conditions. There is some evidence for aggressive fluid therapy with Hartmann's solution in those with severe disease. However, the rate and volume of fluid therapy is still debated for those with mild or moderate disease. Hence, high-quality randomised controlled trials (RCTs) in fluid therapy for acute pancreatitis are necessary.

For peer review only

Goal-directed fluid therapy (GDFT) is a complex intervention in which intravenous (IV) fluid is given to optimise haemodynamic variables. This is achieved by stroke volume optimisation using a cardiac output monitor. RCTs have shown that GDFT decreases complications and mortality in other situations associated with a systemic inflammatory response (12,13). Most of these trials however involved cardiac output monitoring for GDFT during surgery or in the intensive therapy unit (ITU) and not in a ward-based setting. For GDFT to be beneficial in acute pancreatitis, which involves a cascade of inflammatory events, therapy is likely to be most beneficial if commenced at the earliest opportunity to intervene following the onset of pancreatitis which would equate with the time of admission to hospital. There has been one randomised controlled trial on ward based GDFT in patients with acute pancreatitis comparing the effect of GDFT with Lactated ringers to normal saline on inflammatory response (14). However, the trial was based on optimisation of blood urea nitrogen (BUN) and not cardiac output parameters and failed to show a reduction in the inflammatory response or improved clinical outcomes. Whilst other endpoints such as heart rate (HR), urine output (UO), haematocrit levels and central venous pressures have been suggested for fluid therapy in acute pancreatitis, it is the optimisation of intravascular volume with fluid therapy guided by cardiac output measures that has been previously shown to be effective in decreasing complications and mortality in major surgery (15,16). There is currently no RCT investigating

the role of ward based GDFT using cardiac output parameters as target in patients with acute pancreatitis.

With the development of non-invasive cardiac output monitors such as Cheetah NICOM™ (Cheetah Medical Ltd, Maidenhead, Berkshire, UK), it is possible to measure the cardiac output in a ward setting (17). Ward-based GDFT has the potential to decrease the early inflammatory response and hence complications related to acute pancreatitis which might result in decreased mortality and improved health-related quality of life (HRQoL). Reduced inflammation would also lead to a reduced intensive care unit (ICU) and hospital stay providing significant cost savings to the NHS, employers and care providers. This two-centre RCT will aim to assess the feasibility of guiding the initial 48 hours of IV fluid administration in patients with acute pancreatitis using ward-based GDFT.

Methods

Study design and setting

The GAP trial has been designed in accordance with the SPIRIT guidelines as a two centre, feasibility RCT (18). The study will primarily investigate the ability to recruit patients at the selected sites to a feasibility RCT of ward-based GDFT versus standard fluid therapy in patients with acute pancreatitis, the rate of withdrawal from GDFT protocol, and the reasons for withdrawal from GDFT protocol. We will also assess the safety and practicality of ward-based GDFT and collect outcome measures which can be evaluated as end points for a subsequent multi-centre study on efficacy. Indicative costs will be collected to inform a subsequent cost effectiveness study. The trial outline is illustrated in Figure 1 and the schedule of enrolment, interventions and assessments is shown in Table 1.

Participants

For peer review only

Adults (>16 years) admitted as an emergency with acute pancreatitis will be included. Acute pancreatitis must be confirmed by the international consensus criteria (table 2) (19). The exclusion criteria will be patients transferred for the management of complications of acute pancreatitis, requiring immediate admission to the ICU, chronic pancreatitis in whom an acute exacerbation cannot be confirmed, a history of cardiac failure in the past three months and those unable to provide fully informed consent.

Recruitment

Patients referred from the emergency department for admission through the emergency surgical team will be screened by the general surgical registrar on-call. Due to the time-dependant nature of this trial, similar to most trauma and emergency surgery studies, a summarised and abbreviated patient information sheet (PIS) will be provided for patients initially on arrival and they will be subsequently provided with an in-depth version. Informed consent will be obtained by a member of the clinical or research team trained in Good Clinical Practice (GCP) (20). The trial nursing staff responsible for instituting the trial intervention will be contacted within four hours of diagnosis. Intervention or standard of care will commence within six hours of the diagnosis for the next 48 hours of inpatient stay. Since the primary goal of this study is to assess the feasibility of delivering *ward-based* GDFT, patients requiring escalation of care and admission to ICU during the intervention will only have continued cardiac output monitoring until completion of the 48 hour period.

Randomisation

This will be performed using 'Sealed Envelope', an internet-based randomisation system (www.sealedenvolpe.com). Eligible patients who have consented to take part in the trial will be 1:1 randomised to ward based GDFT or standard care, stratified by site on admission, prior to ward transfer. The trial research nurse responsible (unblinded) will log into the database and create randomisation codes for both groups online. Information on randomisation will be stored in the case report form (CRF).

For peer review only

Intervention

GDFT will be carried out for 48 hours. It can take up to 48 hours for the severity of pancreatitis to manifest and is considered the 'golden' period for interventions that may decrease severity (21). GDFT will be based on a standard algorithm which uses the stroke volume (SV) derived from non-invasive cardiac output monitoring using the Cheetah NICOM™ (figure 2). The fluid administration regimen will be as follows: maintenance fluid should be administered at 1.5 ml/kg/hr (based on ideal body weight) using a balanced crystalloid solution. On admission SV is recorded and an initial bolus of 250ml of IV fluid (balanced electrolyte solution) is given over 5 to 10 minutes. If there is a sustained rise in SV of greater than 10% for 15 minutes or more, this indicates fluid responsiveness and a repeat 250ml bolus will be given. If there is not a rise in SV of greater than 10% then the patient is deemed fluid unresponsive and no further fluid boluses are administered. SV monitoring continues four hourly and if it decreases by more than 10% a further fluid bolus is administered as above.

Control (standard care group)

The choice of fluid type, volume and rate of administration on the first 48 hours of admission for patients randomised to the control arm will be decided by the clinical team caring for the patient. Cardiac output monitoring variables will be measured every 4 hours on the standard care arm during the first 48 hours, however, the results will be blinded to the clinical team. The choice of fluid type, volume and rate will be recorded in detail. All other clinical, biochemical and HRQoL outcome measures will also be recorded for standard of care patients as outlined below in 'outcome measures' section.

The Cheetah NICOM™ device (Cheetah Medical Ltd, Maidenhead, Berkshire, UK) is a CE marked device has been purchased for this study and training has been arranged for all staff on the equipment and the associated fluid administration algorithm which will be a key component of the trial. The research team will follow hospital policy on signals for ICU outreach

For peer review only - http://bmjopen.bmj.com/site/about/guidelines.xhtml

reviews and escalation of care to ICU. NICOM monitoring and outcome measures will be recorded in ICU for both control and GDFT arms.

Blinding

It will not be possible to blind the research or clinical team during the first 48 hours of the study. However, the participants, outcome assessors of health-related quality of life, and statisticians will be blinded to the groups. Patient blinding will be aided by NICOM monitoring of both intervention and control groups but performing GDFT in the intervention group alone. NICOM data from the control group will be collected at the same time points as the intervention group and will not be available to the treating clinicians.

Outcome measures

The primary outcome of the trial will be an assessment of feasibility. In particular, the ability to identify and recruit patients at the selected sites to a study of acute pancreatitis. A recruitment rate of at least 30% over 17-month trial period will be deemed as successful. We will also assess the availability of study team for a condition presenting as an emergency, ability to randomise and commence ward GDFT within 6 hours of admission, completion rate of 48 hours of GDFT, rate of withdrawal (less than 20%) from GDFT protocol, reasons for withdrawal from GDFT protocol and proportion of complications in the two groups.

Several secondary clinical, surgical, biochemical, safety and quality of life outcomes will also be collected. Mortality will be assessed as well. Clinical outcomes will include severity of pancreatitis as assessed by Glasgow score, proportion of patients with severe acute pancreatitis, necrotising pancreatitis, infected pancreatic necrosis, requiring ITU stay, requiring renal replacement therapy, requiring ventilation, requiring surgical interventions for complications related to pancreatitis and incidence of positive blood cultures. Resource use data for health economic analysis on length of hospital stay, length of ICU stay, and number of days ventilated, time to return to pre-pancreatitis activities, number of work days lost (in

For peer review only

those who work), and costs (NHS and personal social services (PSS) perspectives) will be collected. Health-related quality of life (HRQoL) will be assessed using the well validated EQ5D questionnaire on admission as baseline and subsequently on day 7, day 30 and day 90. All complications of pancreatitis including treatment-related adverse events (post-radiological and surgical complications as classified by Clavien-Dindo classification) and serious adverse events related to intervention will be recorded up to discharge and at follow-up. Routine biochemical tests performed as part of clinical care including blood gases, liver function tests, clotting profile, renal function, amylase, full blood count and C-reactive protein (CRP) will be recorded on admission and daily up to two weeks and bi-weekly up to discharge. In addition, serum samples will be collected from participants in both groups on admission, 12, 24- and 48-hour time points and stored at -80 degrees for future analysis of biomarkers in acute pancreatitis.

Follow-up

Participants will be followed for 90 days. All clinical and HRQoL outcomes will be measured up to discharge and specifically at 30 days and 90 days by face-to-face or telephone follow-up. CRP will be measured at 24 hours and 48 hours and every 24 hours during the hospital stay as per routine clinical practice. Clinical outcomes on follow-up include cumulative hospital and intensive care unit stay including re-admissions, pre-defined complications including organ failure, sever pancreatitis, infected pancreatic necrosis, surgical interventions, development of sepsis, pulmonary oedema or acute respiratory distress syndrome as well as mortality. Resource use data including medications, planned and unplanned tests and procedures and use of allied healthcare professionals (e.g. physiotherapy, occupational therapy). HRQoL will be assessed using a validated questionnaire (EQ-5D).

Sample size calculation

Formal sample size calculations are not appropriate for a feasibility study, hence a convenient sample size of 50 patients, randomised equally to the two groups, has been chosen. Sample

For peer review only

sizes between 24 and 50 have been recommended to estimate the standard deviation required for a sample size calculation for a subsequent large RCT aimed at evaluating the cost-effectiveness of GDFT (22).

Patient and Public Involvement

Patients involved in the trial preparation were surprised that there had been no progress in effective treatment for acute pancreatitis and felt that new therapeutic approaches were required. Some had experienced late complications and felt that better early treatment may prevent these late complications. The trial protocol was presented and discussed with patients to ensure that patient centred outcomes such as survival and quality of life were appropriately measured, and advice was sought on the conduct of the study. The burden of the intervention was assessed by patient representatives and was thought to be ethical and appropriate. The outcomes of the study will be disseminated to the participants after review and formulation by our study patient representative. We wish to thank our patient representatives for their contribution and participation in this process.

Trial management structure

A Trial Management Group (TMG) chaired by the Chief Investigator and comprising of leads at different sites, ICU research nurses, the surgical team representative, research fellows, statistician, qualitative researchers and health economist will meet fortnightly to discuss trial progress and address issues in recruitment and delivering the intervention. Trial progress will be reported to independent Data Monitoring Committee (DMC) comprising of experts in acute pancreatitis, medical statistics and clinical trials every 6 months.

All serious complications and any mortalities will be immediately referred to the sponsor and reported to the trial management group and independent data monitoring committee. The trial will be stopped in case of two or more treatment related suspected unexpected serious adverse reactions (SUSARs),

Data collection and analysis

For peer review only

Recruitment data which includes number of eligible patients presenting to the emergency department, number of patients screened, number of patients consenting, and number of patients randomised is recorded in a recruitment log in the emergency department. A CONSORT diagram will be presented to provide a detailed description of participant numbers at each time point during the trial (23). A paper CRF (Case Report Form) is created for each participant enrolled into the study. The data collected on the paper CRF is then transferred to a secure online database using the RedCap (Research Electronic Data Capture) platform.

Since this is a feasibility study, all analyses other than recruitment rate and withdrawal rates should be considered exploratory. The two groups will be compared to ensure they have similar baseline characteristics (table 3) using means and standard deviations or medians and inter-quartile ranges for continuous variables, as appropriate, and frequency counts and percentages for categorical variables.

For the primary outcome, i.e., feasibility, the proportion of patients who consent to be randomised and the rate of withdrawal from GDFT protocol will be presented with a 95% confidence interval. The median number of complications, graded by the Clavien-Dindo classification, in each group will be presented. The proportions of people with complications between the two groups will be presented and compared using appropriate statistical test and 95% confidence interval.

For the secondary outcomes, amongst patients who participate in the trial, all clinical and surgical outcome measures will be presented for each group separately using the mean, standard deviation, median, minimum and maximum for continuous outcomes and using frequencies and proportions for categorical variables.

For peer review only

Quality of life will also be summarised for each group using mean profile plots over time. The mean difference in quality of life scores between the two groups at 7 days up will be presented with a 95% confidence interval.

Kaplan-Meier survival estimates and plots will be presented to compare survival rates between the two groups.

All other secondary outcomes collected over time will be summarised for each group using mean profile plots. Mean differences for continuous outcomes and difference in proportion for binary outcomes shall be presented, with appropriate 95% confidence intervals, at 30 and 90 days.

The number and nature of adverse events shall be reported for each group. No formal comparisons between the groups will be made and no hypothesis tests will be carried out. The results will inform us how sensitive the outcome measures are and, along with other information, will be used to determine the primary outcome of a subsequent large RCT. The results will also inform a sample size calculation for the primary outcome chosen.

Assessment of feasibility

This is a feasibility study and one aspect of this feasibility evaluation is the ability to recruit patients with acute pancreatitis into a trial of this nature. According to hospital episode statistics, an in-house review of admissions for acute pancreatitis to the Royal Free Hospital over the last three years showed 107 cases per year. After excluding patients who were transferred from another hospital for tertiary care, those who required immediate ICU admission, those with chronic pancreatitis in whom an acute exacerbation could not be confirmed by internal consensus criteria, current or past cardiac failure, or unable to provide fully informed consent, 80 patients per year would be eligible for this trial from a single centre. With a second centre we would anticipate 120 suitable patients per year. We anticipate

For peer review only

recruiting at least 30% of potential participants which equates to 36 participants annually and a recruitment time of 17 months to recruit the 50 patients.

For feasibility assessment, a recruitment log will be used to collect information on suitability and consent rate as well as availability of trials team for GDFT, consent, failure to commence ward GDFT, withdrawal with reasons and failure to complete the study. In case of lower than anticipated recruitment at six months, we will perform the following; a) analyse the results of the qualitative research earlier and identify the reasons for poor recruitment (and take actions to resolve the problems) if we get the anticipated number of patients screened but the recruitment rate is lower than anticipated, b) recruitment of additional centres if we do not get the anticipated number of potential participants screened but the recruitment rate is at least as good as the anticipated rate c) a combination of the above if the number of potential participants screened and the recruitment rate are lower than anticipated.

We will explore the reasons for participation and non-participation of eligible patients and patients' and clinicians' acceptability of the trial to assist in optimisation of recruitment strategies employed for the definitive trial. Non-participation can be related to how the clinical trial is presented to the patient, and how the patient assimilates this information. It is therefore important to understand how patients perceive information about potential participation and their experiences of receiving information relating to the trial. Interviews with a sample of eligible patients will explore patient perspectives of treatment, their understanding of the two treatments, reasons for taking part or refusing the trial, and the acceptability of randomisation between the procedures. Interviews with clinical staff will explore their views about the trial, clinical equipoise, and their understanding of the recruitment challenges. Semi-structured interviews will be informed by a topic guide developed in conjunction with the trial management group which includes patient representatives. Patient information sessions (recruiter meetings) will be audio recorded to examine how information is presented, and identify issues potentially affecting trial recruitment. Patient information sessions will be analysed at an early

For peer review only - http://bmjopen.bmj.com/site/about/guidelines.xhtml

stage if there is poor recruitment and will inform the development of any additional training materials for recruitment for the definitive trial.

Progress to full trial

The criteria to progress to a subsequent full trial will be determined quantitatively as a) consent rate of at least 30%, the ability to recruit 50 patients to the study at the two sites over 17 months, b) GDFT can be successfully performed within 6 hours of diagnosis of acute pancreatitis and can be continued until at least 48 hours after admission in a minimum of 80% of participants randomised to GDFT, and c) the complication rate in the intervention group is not more than 10% higher than that of the control group at 90 days. A cut off of 30% has been chosen for recruitment as this would be an achievable target in the definitive trial. A lower recruitment rate may indicate a lack of acceptability among clinicians and/or patients to participate in the definitive trial. In addition, a recruitment rate less than 30% is likely to make the subsequent definitive trial very expensive. A less than 30% recruitment may also highlight issues related to generalisability of the results, indicating a reduction in value of the subsequent definitive trial in terms of applicability in NHS.

Ethics and Dissemination

The trial protocol (v2) has been reviewed and approved by the London Central Research Ethics Committee (REC ref: 17/LO/1235, project ID: 221872). The results of this study will be presented to the national and international meetings with interest in the management of acute pancreatitis and prepared for publication in peer-reviewed journals with a readership in general surgery and critical care.

Discussion

Acute pancreatitis is an inflammatory disease associated with a high mortality rate (24). Adequate fluid resuscitation has been identified as a key intervention in prevention of systemic complications, however, the debate over the type, rate, or amount of intravenous fluids to be

For peer review only

administered is ongoing (25). With the advent of non-invasive cardiac output monitoring (NICOM) devices, delivering haemodynamic guided fluid replacement is now possible in patients admitted to the general ward. The GAP trial is the first ward-based RCT of GDFT in acute pancreatitis. The trial will assess feasibility and safety of delivering this intervention in the ward and if successful, will progress to a multicentre trial to assess efficacy and cost-effectiveness.

Trial Status

The GAP trial opened for recruitment on 8th January 2018 for a period of 18 months.

List of Abbreviations

- C-Reactive Protein (CRP)
- Data Monitoring Committee (DMC)
- GDFT in Acute Pancreatitis (GAP)
- Goal Directed Fluid Therapy (GDFT)
- Health-Related Quality of Life (HRQoL)
- Intensive Care Unit (ICU)
- Intravenous (IV)
- National Health System (NHS)
- Non-Invasive Cardiac Output Monitor (NICOM)
- Patient Information Sheet (PIS)
- Personal Social Services (PSS)
- Randomized Control Trial (RCT)
- Stroke Volume (SV)
- Suspected Unexpected Serious Adverse Reactions (SUSAR)
- Trial Management Group (TMG)

Declarations

For peer review only

Ethical approval and consent to participate

The trial protocol (v2) has been reviewed and approved by the London Central Research Ethics Committee (REC ref: 17/LO/1235, project ID: 221872). Informed consent will be obtained from eligible patients after screening by a member of the research or clinical team trained in Good Clinical Practice (GCP). The trial has been registered on ISRCTN (ISRCTN 36077283) on 09 April 2018 (http://www.isrctn.com/ISRCTN36077283).

Consent for publication

Not applicable.

Availability of data and materials

Data from this trial will be made available after analysis and publication upon reasonable request from the corresponding author.

Competing interests

The authors declare that they have no competing interests.

Funding

This study is funded by the National Institute for Health Research (NIHR) Research for Patient Benefit Programme (RfPB) (Grant Reference Number PB-PG-0815-20002). This paper presents independent research funded by the (NIHR) under its Research for Patient Benefit (RfPB) Programme (Grant Reference Number PB-PG-0815-20002). The views expressed are those of the author(s) and not necessarily those of the NHS, the NIHR or the Department of Health.

Author contributions

For peer review only

FF manuscript preparation, mechanistic components of protocol design, trial and database management, FS manuscript preparation, trial management, FR statistical analysis plan, KG major contribution to design and funding application, DM protocol design of GDFT regimes, trial management, SS initial protocol developments and ethical approvals, CE, MC, OS, GA, MMcN and HF trial research nurses trained in delivering GDFT, data collection, JS health economic analysis design, MV participant enrolment planning through emergency surgery , BD principal investigator and grant holder, overall trial management, study design and review of manuscript. All authors read and approved the final manuscript.

Acknowledgements

We are grateful for the support of our emergency surgical colleagues Mr Olegunju Ogunbiyi, Mr Jonathan Knowles, Mr Rovan D'Souza, Mr Colin Hart, Mr Ahmad Hamad, the surgical registrars Ms Tanzeela Gala, Ms Karen Bosch, Ms Aliza Abeles, Mr Guillaume Lafaurie, Mr Tom Hanna, Mr Stephanos Pericleous, our emergency department lead Dr Jonathan Costello, without whom the conduct of this trial would not be possible. We wish to thank our patient representative Ms Lucy Cotterell for their contributions. We gratefully acknowledge the financial support of the Wellington hospital, London, in their HPB Surgery Fellowship funding for Mr Farid Froghi.

For peer review only

References

1. Roberts SE, Akbari A, Thorne K, Atkinson M, Evans PA. The incidence of acute pancreatitis: Impact of social deprivation, alcohol consumption, seasonal and demographic factors. Aliment Pharmacol Ther [Internet]. Wiley-Blackwell; 2013 Sep [cited 2018 Sep 5];38(5):539–48. Available from: http://www.ncbi.nlm.nih.gov/pubmed/23859492

2. Working Party of the British Society of Gastroenterology, Association of Surgeons of Great Britain and Ireland, Pancreatic Society of Great Britain and Ireland, Association of Upper GI Surgeons of Great Britain and Ireland. UK guidelines for the management

of acute pancreatitis. Gut [Internet]. 2005;54(suppl 3):iii1-iii9. Available from: http://gut.bmj.com/content/54/suppl_3/iii1

3. Párniczky A, Kui B, Szentesi A, Balázs A, Szûcs Á, Mosztbacher D, et al. Prospective, Multicentre, Nationwide Clinical Data from 600 Cases of Acute Pancreatitis. PLoS One. 2016;11(10):1–19.

4. Leung PS, Ip SP. Pancreatic acinar cell: Its role in acute pancreatitis. Vol. 38, International Journal of Biochemistry and Cell Biology. 2006. p. 1024–30.

5. Andersson R, Andersson B, Andersson E, Axelsson J, Eckerwall G, Tingstedt B. Acute pancreatitis - From cellular signalling to complicated clinical course. Hpb. 2007;9(6):414–20.

6. ’tHart NA, Leuvenink HGD, Ploeg RJ. New solutions in organ preservation. Transplant Rev [Internet]. W.B. Saunders; 2002 Jul 1 [cited 2017 Dec 11];16(3):131–41. Available from: http://www.sciencedirect.com/science/article/pii/S0955470X02800027

7. De Beaux AC, Palmer KR, Carter DC. Factors influencing morbidity and mortality in acute pancreatitis; an analysis of 279 cases. Gut [Internet]. BMJ Publishing Group; 1995 Jul [cited 2018 Sep 14];37(1):121–6. Available from: http://www.ncbi.nlm.nih.gov/pubmed/7672660

8. Vege SS, Gardner TB, Chari ST, Munukuti P, Pearson RK, Clain JE, et al. Low Mortality and High Morbidity in Severe Acute Pancreatitis Without Organ Failure: A Case for Revising the Atlanta Classification to Include “Moderately Severe Acute Pancreatitis.” Am J Gastroenterol [Internet]. Nature Publishing Group; 2009 Mar 3 [cited 2018 Sep 14];104(3):710–5. Available from: http://www.nature.com/articles/ajg200877

9. Moggia E, Koti R, Belgaumkar AP, Fazio F, Pereira SP, Davidson BR, et al. Pharmacological interventions for acute pancreatitis. Vol. 2017, Cochrane Database of Systematic Reviews. 2017. p. 1–14.

10. Iqbal U, Anwar H, Scribani M. Ringer’s Lactate vs Normal Saline in Acute Pancreatitis: A Systematic Review and meta- analysis.

For peer review only

11. Szentesi A, Tóth E, Bálint E, Fanczal J, Madácsy T, Laczkó D, et al. Analysis of research activity in gastroenterology: Pancreatitis is in real danger. PLoS One [Internet]. Public Library of Science; 2016 [cited 2019 Apr 11];11(10):e0165244. Available from: http://www.ncbi.nlm.nih.gov/pubmed/27776171

12. Pearse RM, Harrison DA, MacDonald N, Gillies MA, Blunt M, Ackland G, et al. Effect of a Perioperative, Cardiac Output–Guided Hemodynamic Therapy Algorithm on Outcomes Following Major Gastrointestinal Surgery. JAMA [Internet]. American Medical Association; 2014 Jun 4 [cited 2018 Oct 11];311(21):2181. Available from: http://jama.jamanetwork.com/article.aspx?doi=10.1001/jama.2014.5305

13. Hamilton MA, Cecconi M, Rhodes A. A Systematic Review and Meta-Analysis on the Use of Preemptive Hemodynamic Intervention to Improve Postoperative Outcomes in Moderate and High-Risk Surgical Patients. Anesth Analg [Internet]. 2011 Jun [cited 2016 Aug 27];112(6):1392–402. Available from: http://content.wkhealth.com/linkback/openurl?sid=WKPTLP:landingpage&an=00000539-201106000-00027

14. Wu BU, Hwang JQ, Gardner TH, Repas K, Delee R, Yu S, et al. Lactated Ringer's Solution Reduces Systemic Inflammation Compared With Saline in Patients With Acute Pancreatitis. Clin Gastroenterol Hepatol. 2011;9(8):710–7.

15. Rivers EP, Ander DS, Powell D. Central venous oxygen saturation monitoring in the critically ill patient. Curr Opin Crit Care. 2001;7(3):204–11.

16. Bortolotti P, Saulnier F, Colling D, Redheuil A, Preau S. New tools for optimizing fluid resuscitation in acute pancreatitis. World J Gastroenterol [Internet]. Baishideng Publishing Group Inc; 2014 Nov 21 [cited 2018 Oct 11];20(43):16113–22. Available from: http://www.ncbi.nlm.nih.gov/pubmed/25473163

17. Dunham CM, Chirichella TJ, Gruber BS, Ferrari JP, Martin JA, Luchs BA, et al. Emergency department noninvasive (NICOM) cardiac outputs are associated with trauma activation, patient injury severity and host conditions and mortality. J Trauma Acute Care Surg. 2012;73(2):479–85.

For peer review only

18. Chan A-W, Tetzlaff JM, Gøtzsche PC, Altman DG, Mann H, Berlin JA, et al. SPIRIT 2013 explanation and elaboration: guidance for protocols of clinical trials. BMJ [Internet]. BMJ Group; 2013 Jan 8 [cited 2016 Oct 25];346:e7586. Available from: http://www.ncbi.nlm.nih.gov/pubmed/23303884

19. Tenner S, Baillie J, Dewitt J, Vege SS. American college of gastroenterology guideline: Management of acute pancreatitis. Am J Gastroenterol. Nature Publishing Group; 2013;108(9):1400–15.

20. Good Clinical Practice (GCP) [Internet]. [cited 2018 Sep 14]. Available from: https://www.nihr.ac.uk/our-faculty/clinical-research-staff/learning-and-development/national-directory/good-clinical-practice/

21. Fisher JM, Gardner TB. The "Golden Hours" of Management in Acute Pancreatitis. Am J Gastroenterol [Internet]. Nature Publishing Group; 2012 Aug 3 [cited 2018 Oct 11];107(8):1146–50. Available from: http://www.nature.com/articles/ajg201291

22. Sim J, Lewis M. The size of a pilot study for a clinical trial should be calculated in relation to considerations of precision and efficiency. J Clin Epidemiol [Internet]. Elsevier; 2012 Mar 1 [cited 2018 Sep 15];65(3):301–8. Available from: http://www.ncbi.nlm.nih.gov/pubmed/22169081

23. Moher D, Schulz KF, Altman DG. The CONSORT statement: revised recommendations for improving the quality of reports of parallel-group randomised trials. Lancet [Internet]. Elsevier; 2001 Apr 14 [cited 2018 Dec 22];357(9263):1191–4. Available from: https://www.sciencedirect.com/science/article/pii/S0140673600043373?via%3Dihub

24. Bank S, Singh P, Pooran N, Stark B. Evaluation of Factors That Have Reduced Mortality From Acute Pancreatitis Over the Past 20 Years. J Clin Gastroenterol [Internet]. 2002;35(1). Available from: https://journals.lww.com/jcge/Fulltext/2002/07000/Evaluation_of_Factors_That_Have_Reduced_Mortality.12.aspx

25. Aggarwal A, Manrai M, Kochhar R. Fluid resuscitation in acute pancreatitis. World J

For peer review only

575 Gastroenterol [Internet]. Baishideng Publishing Group Inc; 2014 Dec 28 [cited 2018
576 Sep 15];20(48):18092–103. Available from:
577 http://www.ncbi.nlm.nih.gov/pubmed/25561779

578

579

For peer review only

Table 1. Schedule of enrolment, interventions and assessments

Procedures	Enrolment	Allocation	Post-allocation								Close-out
**Time point**	**-t0**	**0**	**0 hrs**	**12 hrs**	**24 hrs**	**48 hrs**	**discharge**	**7 days**	**30 days**	**90 days**	
**ENROLMENT**											
Eligibility screen	x										
PIS given	x										
Informed consent	x										
Registration	x										
Allocation		x									
**INTERVENTION**											
GDFT (intervention)			◆———	———	———	———◆					
Standard care (control)			◆———	———	———	———◆					
ASSESSMENTS											
NICOM Monitoring			◆———	———	———	———◆					
Blood tests			x	x	x	x					
HRQoL			x					x	x	x	x
Complications			x	x	x	x	x		x	x	x
Other outcomes			x	x	x	x	x		x	x	x

For peer review only

Table 2. International consensus criteria for acute pancreatitis

Acute pancreatitis diagnosis confirmed with two of the following three features:
• Abdominal pain consistent with acute pancreatitis (acute onset of a persistent, severe, epigastric pain often radiating to the back)<br>• Serum amylase or lipase activity at least three times greater than the upper limit of normal.<br>• Characteristic findings of acute pancreatitis on contrast-enhanced computed tomography (CECT) and less commonly magnetic resonance imaging (MRI) or transabdominal ultrasonography.

For peer review only

Table 3. Baseline characteristics of patients

Age
Sex
Ethnicity
BMI
Time since onset of symptoms
Presumed cause of pancreatitis
Comorbidities
Baseline observations
Baseline renal function
Baseline blood gas parameters
Glasgow severity score on admission
Intravenous fluids prior to intervention

For peer review only

Figure Legends:

Figure 1. Trial flow diagram
Patients are screened by the medical and surgical team in the emergency department for eligibility and referred for consent and registration. The trial nurse or surgical team provide trial patient information sheets (abbreviated and full versions) and gain informed consent. Trial nurses then randomise the patient to either GDFT or standard care and commence NICOM monitoring for 48 hours. Follow-up will be at the point of discharge, 30 days and 90 days.

Figure 2. Goal-directed fluid therapy (intervention) protocol
Flow diagram of SV optimisation with maintenance fluid administration. Patients randomised to GDFT will receive a 1.5ml/kg/hr of intravenous crystalloid maintenance fluid and four hourly visits from the trial nurses. At the first 4-hour visit, a bolus of 250mL crystalloid over 5-10mins will be administered. A SV rise of >10% would indicate fluid responsiveness and a further cycle of fluid bolus is administered until there is no SV rise >10%. At the next 4 hourly visit if SV has dropped by >10% the cycle is repeated.

For peer review only

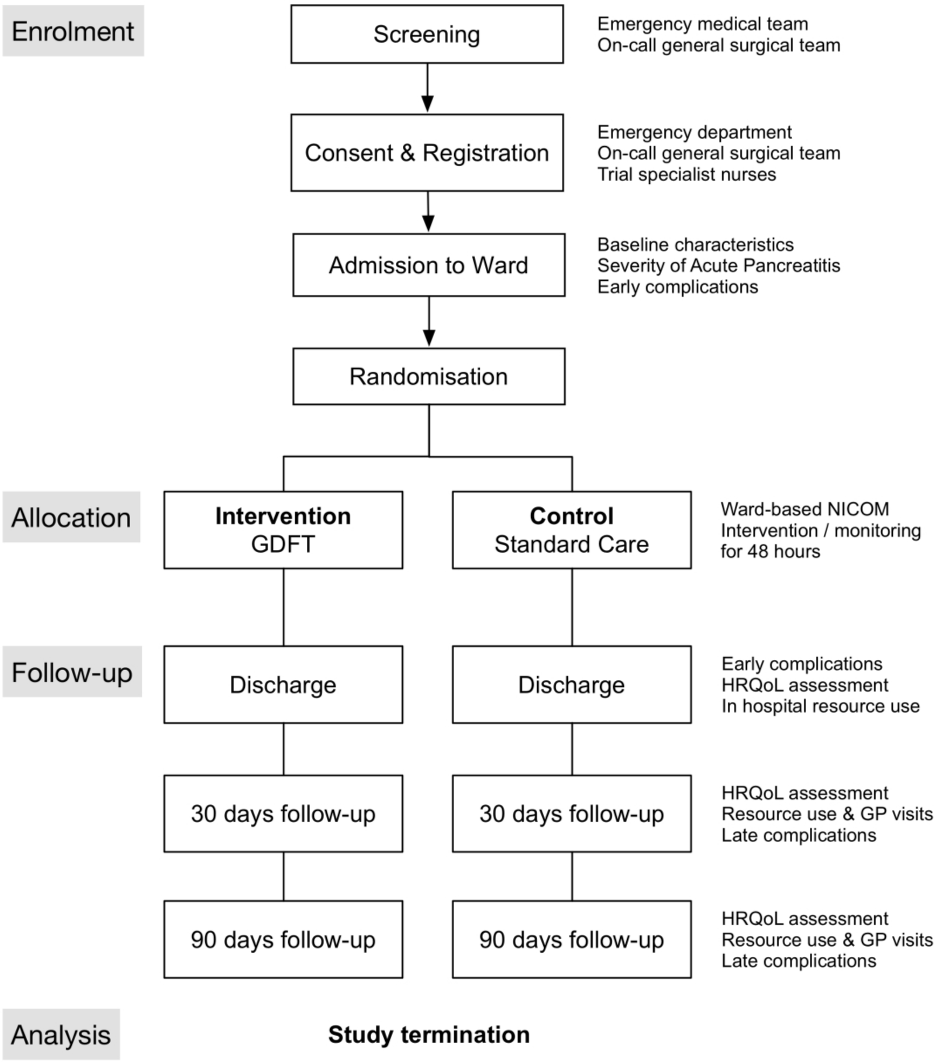

Figure 1 Trial flow diagram

207x234mm (300 x 300 DPI)

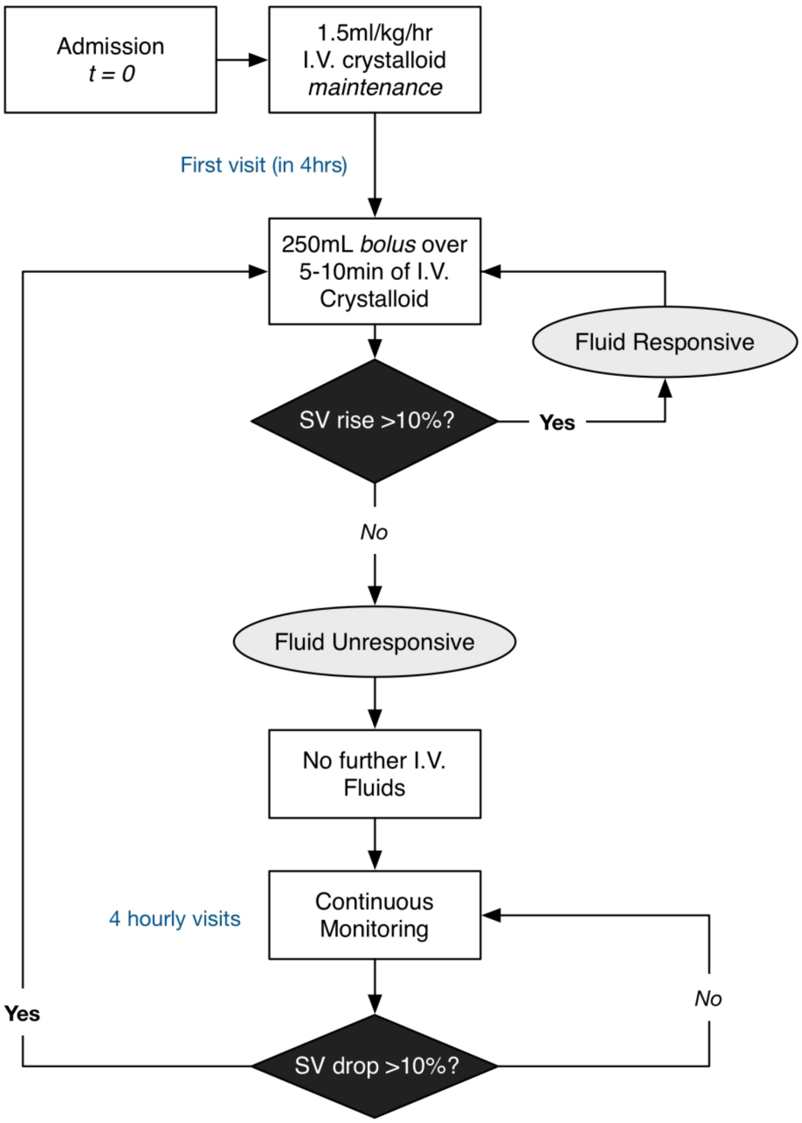

Figure 2 Goal-directed fluid therapy protocol

155x216mm (300 x 300 DPI)

Reporting checklist for protocol of a clinical trial.

Based on the SPIRIT guidelines.

Instructions to authors

Complete this checklist by entering the page numbers from your manuscript where readers will find each of the items listed below.

Your article may not currently address all the items on the checklist. Please modify your text to include the missing information. If you are certain that an item does not apply, please write "n/a" and provide a short explanation.

Upload your completed checklist as an extra file when you submit to a journal.

In your methods section, say that you used the SPIRIT reporting guidelines, and cite them as:

Chan A-W, Tetzlaff JM, Altman DG, Laupacis A, Gøtzsche PC, Krleža-Jerić K, Hróbjartsson A, Mann H, Dickersin K, Berlin J, Doré C, Parulekar W, Summerskill W, Groves T, Schulz K, Sox H, Rockhold FW, Rennie D, Moher D. SPIRIT 2013 Statement: Defining standard protocol items for clinical trials. Ann Intern Med. 2013;158(3):200-207

		Reporting Item	Page Number
Title	#1	Descriptive title identifying the study design, population, interventions, and, if applicable, trial acronym	1
Trial registration	#2a	Trial identifier and registry name. If not yet registered, name of intended registry	2
Trial registration: data set	#2b	All items from the World Health Organization Trial Registration Data Set	2
Protocol version	#3	Date and version identifier	16
Funding	#4	Sources and types of financial, material, and other support	17
Roles and responsibilities: contributorship	#5a	Names, affiliations, and roles of protocol contributors	17
Roles and responsibilities:	#5b	Name and contact information for the trial sponsor	17

For peer review only

sponsor contact information			
Roles and responsibilities: sponsor and funder	#5c	Role of study sponsor and funders, if any, in study design; collection, management, analysis, and interpretation of data; writing of the report; and the decision to submit the report for publication, including whether they will have ultimate authority over any of these activities	17
Roles and responsibilities: committees	#5d	Composition, roles, and responsibilities of the coordinating centre, steering committee, endpoint adjudication committee, data management team, and other individuals or groups overseeing the trial, if applicable (see Item 21a for data monitoring committee)	17, 10
Background and rationale	#6a	Description of research question and justification for undertaking the trial, including summary of relevant studies (published and unpublished) examining benefits and harms for each intervention	4-6
Background and rationale: choice of comparators	#6b	Explanation for choice of comparators	4-6 / 8
Objectives	#7	Specific objectives or hypotheses	6
Trial design	#8	Description of trial design including type of trial (eg, parallel group, crossover, factorial, single group), allocation ratio, and framework (eg, superiority, equivalence, non-inferiority, exploratory)	6
Study setting	#9	Description of study settings (eg, community clinic, academic hospital) and list of countries where data will be collected. Reference to where list of study sites can be obtained	6
Eligibility criteria	#10	Inclusion and exclusion criteria for participants. If applicable, eligibility criteria for study centres and individuals who will perform the interventions (eg, surgeons, psychotherapists)	6
Interventions: description	#11a	Interventions for each group with sufficient detail to allow replication, including how and when they will be administered	8
Interventions: modifications	#11b	Criteria for discontinuing or modifying allocated interventions for a given trial participant (eg, drug dose change in response to harms, participant request, or improving / worsening disease)	8
Interventions:	#11c	Strategies to improve adherence to intervention protocols, and any	8

For peer review only

adherance		procedures for monitoring adherence (eg, drug tablet return; laboratory tests)	
Interventions: concomitant care	#11d	Relevant concomitant care and interventions that are permitted or prohibited during the trial	8
Outcomes	#12	Primary, secondary, and other outcomes, including the specific measurement variable (eg, systolic blood pressure), analysis metric (eg, change from baseline, final value, time to event), method of aggregation (eg, median, proportion), and time point for each outcome. Explanation of the clinical relevance of chosen efficacy and harm outcomes is strongly recommended	9
Participant timeline	#13	Time schedule of enrolment, interventions (including any run-ins and washouts), assessments, and visits for participants. A schematic diagram is highly recommended (see Figure)	Table 1
Sample size	#14	Estimated number of participants needed to achieve study objectives and how it was determined, including clinical and statistical assumptions supporting any sample size calculations	10
Recruitment	#15	Strategies for achieving adequate participant enrolment to reach target sample size	7
Allocation: sequence generation	#16a	Method of generating the allocation sequence (eg, computer-generated random numbers), and list of any factors for stratification. To reduce predictability of a random sequence, details of any planned restriction (eg, blocking) should be provided in a separate document that is unavailable to those who enrol participants or assign interventions	7
Allocation concealment mechanism	#16b	Mechanism of implementing the allocation sequence (eg, central telephone; sequentially numbered, opaque, sealed envelopes), describing any steps to conceal the sequence until interventions are assigned	7
Allocation: implementation	#16c	Who will generate the allocation sequence, who will enrol participants, and who will assign participants to interventions	7
Blinding (masking)	#17a	Who will be blinded after assignment to interventions (eg, trial participants, care providers, outcome assessors, data analysts), and how	9
Blinding (masking):	#17b	If blinded, circumstances under which unblinding is permissible,	9

For peer review only

emergency unblinding		and procedure for revealing a participant's allocated intervention during the trial	
Data collection plan	#18a	Plans for assessment and collection of outcome, baseline, and other trial data, including any related processes to promote data quality (eg, duplicate measurements, training of assessors) and a description of study instruments (eg, questionnaires, laboratory tests) along with their reliability and validity, if known. Reference to where data collection forms can be found, if not in the protocol	11
Data collection plan: retention	#18b	Plans to promote participant retention and complete follow-up, including list of any outcome data to be collected for participants who discontinue or deviate from intervention protocols	11
Data management	#19	Plans for data entry, coding, security, and storage, including any related processes to promote data quality (eg, double data entry; range checks for data values). Reference to where details of data management procedures can be found, if not in the protocol	11
Statistics: outcomes	#20a	Statistical methods for analysing primary and secondary outcomes. Reference to where other details of the statistical analysis plan can be found, if not in the protocol	11-13
Statistics: additional analyses	#20b	Methods for any additional analyses (eg, subgroup and adjusted analyses)	11
Statistics: analysis population and missing data	#20c	Definition of analysis population relating to protocol non-adherence (eg, as randomised analysis), and any statistical methods to handle missing data (eg, multiple imputation)	11
Data monitoring: formal committee	#21a	Composition of data monitoring committee (DMC); summary of its role and reporting structure; statement of whether it is independent from the sponsor and competing interests; and reference to where further details about its charter can be found, if not in the protocol. Alternatively, an explanation of why a DMC is not needed	
Data monitoring: interim analysis	#21b	Description of any interim analyses and stopping guidelines, including who will have access to these interim results and make the final decision to terminate the trial	10
Harms	#22	Plans for collecting, assessing, reporting, and managing solicited and spontaneously reported adverse events and other unintended effects of trial interventions or trial conduct	11

For peer review only

Auditing	#23	Frequency and procedures for auditing trial conduct, if any, and whether the process will be independent from investigators and the sponsor	11
Research ethics approval	#24	Plans for seeking research ethics committee / institutional review board (REC / IRB) approval	16
Protocol amendments	#25	Plans for communicating important protocol modifications (eg, changes to eligibility criteria, outcomes, analyses) to relevant parties (eg, investigators, REC / IRBs, trial participants, trial registries, journals, regulators)	11
Consent or assent	#26a	Who will obtain informed consent or assent from potential trial participants or authorised surrogates, and how (see Item 32)	N/A
Consent or assent: ancillary studies	#26b	Additional consent provisions for collection and use of participant data and biological specimens in ancillary studies, if applicable	N/A
Confidentiality	#27	How personal information about potential and enrolled participants will be collected, shared, and maintained in order to protect confidentiality before, during, and after the trial	11
Declaration of interests	#28	Financial and other competing interests for principal investigators for the overall trial and each study site	16
Data access	#29	Statement of who will have access to the final trial dataset, and disclosure of contractual agreements that limit such access for investigators	16
Ancillary and post trial care	#30	Provisions, if any, for ancillary and post-trial care, and for compensation to those who suffer harm from trial participation	N/A
Dissemination policy: trial results	#31a	Plans for investigators and sponsor to communicate trial results to participants, healthcare professionals, the public, and other relevant groups (eg, via publication, reporting in results databases, or other data sharing arrangements), including any publication restrictions	15
Dissemination policy: authorship	#31b	Authorship eligibility guidelines and any intended use of professional writers	N/A
Dissemination policy: reproducible research	#31c	Plans, if any, for granting public access to the full protocol, participant-level dataset, and statistical code	15
Informed consent	#32	Model consent form and other related documentation given to	Appendix

For peer review only

materials		participants and authorised surrogates	
Biological specimens	#33	Plans for collection, laboratory evaluation, and storage of biological specimens for genetic or molecular analysis in the current trial and for future use in ancillary studies, if applicable	10

The SPIRIT checklist is distributed under the terms of the Creative Commons Attribution License CC-BY-ND 3.0. This checklist can be completed online using https://www.goodreports.org/, a tool made by the EQUATOR Network in collaboration with Penelope.ai

For peer review only

BMJ Open

Ward based Goal-Directed Fluid Therapy (GDFT) in Acute Pancreatitis (GAP trial): study protocol for a feasibility randomised controlled trial

Journal:	*BMJ Open*
Manuscript ID	bmjopen-2018-028783.R2
Article Type:	Protocol
Date Submitted by the Author:	30-Aug-2019
Complete List of Authors:	Froghi, Farid; University College London, Division of Surgery & Interventional Science; Royal Free Hospital, HPB and Liver Transplantation Surgery<br>Soggiu, Fiammetta; Royal Free Hospital, HPB and Liver Transplantation Surgery<br>Ricciardi, Federico; University College London, Statistical Sciences<br>Gurusamy, Kurinchi; University College London, Division of Surgery & Interventional Sciences<br>Martin, Daniel S.; University College London, Division of Surgery & Interventional Sciences; Royal Free Hospital, Critical Care Unit<br>Singh, Jeshika; PHMR Ltd, Health Economics<br>Siddique, Sulman; University College London, Division of Surgery & Interventional Sciences<br>Eastgate, Christine; Royal Free Hospital, Critical Care Unit<br>Ciaponi, Maria; Royal Free Hospital, Critical Care Unit<br>McNeil, Margaret; Royal Free Hospital, Critical Care Unit<br>Filipe, Helder; Royal Free Hospital, Critical Care Unit<br>Schwalowsky-Monks, Otto; Royal Free Hospital, Critical Care Unit<br>Asis, Gretchelle; Royal Free Hospital, Critical Care Unit<br>Varcada, Massimo; Royal Free Hospital, General and Emergency Surgery<br>Davidson, Brian; University College London, Division of Surgery & Interventional Sciences; Royal Free Hospital, HPB and Liver Transplantation Surgery
**Primary Subject Heading**:	Surgery
Secondary Subject Heading:	Gastroenterology and hepatology, Research methods
Keywords:	Acute Pancreatitis, Goal-Directed Fluid Therapy, Fluid Therapy, Cardiac Output

SCHOLARONE™
Manuscripts

Ward based Goal-Directed Fluid Therapy (GDFT) in Acute Pancreatitis (GAP trial): study protocol for a feasibility randomised controlled trial

Farid Froghi[1], Fiammetta Soggiu[1], Federico Ricciardi[5], Kurinchi Gurusamy[1], Daniel Martin[1,2], Jeshika Singh[4], Sulman Siddique[1], Christine Eastgate[2], Maria Ciaponi[2], Margaret McNeil[2], Helder Filipe[2], Otto Schwalowsky-Monks[2], Gretchelle Asis[2], Massimo Varcada[1,3], Brian Davidson[1]

[1]UCL Division of Surgery and Interventional Science, Royal Free Hospital, London

[2]Critical Care Unit, Royal Free Hospital, London

[3]General and Emergency Surgery Department, Royal Free Hospital, London

[4]PHMR, Health Economics, Pricing & Reimbursement, London, United Kingdom

[5]Department of Statistical Science, University College London, London

Corresponding author: Mr Farid Froghi
UCL Division of Surgery & Interventional Sciences
9th Floor, Royal Free Hospital
Pond Street
London NW3 2QG

Email: farid.froghi@nhs.net

Tel: 020 7794 0500

For peer review only

Abstract

Introduction: Acute pancreatitis is an inflammatory disease of the pancreas with high risk of developing multiorgan failure and death. There are no effective pharmacological interventions used in current clinical practice. Maintaining fluid and electrolyte balance is the mainstay of supportive management. Goal directed fluid therapy (GDFT) has been shown to decrease morbidity and mortality in surgical conditions with systemic inflammatory response. There is currently no randomized controlled trial (RCT) investigating the role of GDFT based on cardiac output parameters in patients with acute pancreatitis in the ward setting. A feasibility trial was designed to determine patient and clinician support for recruitment into a RCT of ward-based GDFT in acute pancreatitis, adherence to a GDFT protocol, safety, participant withdrawal, and to determine appropriate endpoints for a subsequent larger trial to evaluate efficacy.

Methods and Analysis: The GDFT in Acute Pancreatitis (GAP) trial is a prospective two-centre, feasibility RCT. Eligible adults admitted with new onset of acute pancreatitis will be enrolled and randomised into ward-based GDFT (n=25) or standard fluid therapy (n=25) within 6 hours from the diagnosis and continuing for the following 48 hours. Cardiac output parameters will be monitored with a non-invasive device (Cheetah NICOM ®; Cheetah Medical Ltd, Maidenhead, Berkshire, UK). The intervention will consist of a protocoled GDFT approach consisting of stroke volume optimisation with crystalloid fluid boluses, while the control group will receive standard care fluid therapy as advised by the clinical team. The primary endpoint is feasibility. Secondary endpoints will include safety of the intervention, complications, mortality, admission to intensive care unit, cost and quality of life.

Ethics and Dissemination: Ethics approved by the London Central Research Ethics Committee (17/LO/1235, project-ID: 221872). The results of this trial will be presented in international conference with interest in general surgery and acute care and published in a peer-reviewed journal.

Trial registration: ISRCTN 36077283 (26-03-2018)

For peer review only

Keywords:

Fluid therapy, Acute Pancreatitis, Cardiac Output, Goal-directed Fluid Therapy

Strengths and limitations of this study

- Adequate fluid therapy is a major determinant of outcomes in Acute Pancreatitis, however, there is no consensus on the rate, type and volume of fluids to be administered. Goal-directed fluid therapy (GDFT) based on stroke volume (SV) optimisation has been used after major surgery with a reduction in complications, however, delivering this intervention on the ward has been challenging without invasive monitoring.
- Novel development of non-invasive cardiac output monitoring (NICOM) technologies allows GDFT to be delivered in the ward based on haemodynamic parameters. This trial is the first GDFT randomised trial in acute pancreatitis using non-invasive cardiac output monitoring in the ward setting for SV optimisation.
- The golden period for intervention in acute pancreatitis is in the early phase of disease to prevent progression to severe inflammatory processes and complications. A major challenge in this trial is recruitment and delivering the intervention in the early hours of unplanned admission which is shared across trials in emergency care.

For peer review only

Introduction

Acute pancreatitis is a sudden onset inflammatory process of the pancreas, with variable involvement of local or remote organ systems. The annual incidence of acute pancreatitis in the UK is approximately 30 per 100,000 of the population (1). This equates to approximately 18,000 people developing acute pancreatitis every year in the UK (1). There has been an increase in the incidence of pancreatitis in the last two decade (2). Gallstones and excessive alcohol are the two main causes for acute pancreatitis in the UK (3). Increasing age, male gender, and lower socioeconomic class are associated with a higher incidence of acute pancreatitis (1).

The clinical manifestation of acute pancreatitis is believed to be caused by activation of inflammatory pathways either directly by the pathologic insult or indirectly by activation of trypsin, a protease which can break down the pancreas (4). The average 60 day mortality associated with acute pancreatitis is 6.4% (1). Deaths occur as a result of a massive fluid extravasation from the inflamed pancreas combined with a severe systemic inflammatory response in the early stage and mainly from local complications in the late stage (5). The systemic complications occurring in the early phase result in poor oxygen delivery to the tissues and include worsening of pre-existing illnesses such as heart or chronic lung disease. Local complications which occur in the later phase include pancreatic pseudocysts, infected collections and pancreatic necrosis. These early and late complications are the major causes of the mortality associated with acute pancreatitis (6,7). They are also largely responsible for the decreased health-related quality of life and loss of work days following acute pancreatitis (8). Thus, acute pancreatitis has a major effect on UK society with its complications having a significant impact on patients, their next of kin, and their employers.

Various pharmacological interventions have been evaluated in acute pancreatitis, but none are in current clinical practice (9). Supportive management in terms of maintenance of fluid and electrolyte balance remains the mainstay in the treatment of acute pancreatitis. Despite

For peer review only

the key importance of fluid therapy, a recent systematic review has highlighted that there is a lack of information on the optimal fluid therapy in acute pancreatitis (9,10). There has also been a decline in research in treatments for acute pancreatitis in general (11).

The clinical presentation of acute pancreatitis varies from mild to severe life-threatening conditions. There is some evidence for aggressive fluid therapy with Hartmann's solution in those with severe disease. However, the rate and volume of fluid therapy is still debated for those with mild or moderate disease. Hence, high-quality randomised controlled trials (RCTs) in fluid therapy for acute pancreatitis are necessary.

Goal-directed fluid therapy (GDFT) is a complex intervention in which intravenous (IV) fluid is given to optimise haemodynamic variables. This is achieved by stroke volume optimisation using a cardiac output monitor. RCTs have shown that GDFT decreases complications and mortality in other situations associated with a systemic inflammatory response (12,13). Most of these trials however involved cardiac output monitoring for GDFT during surgery or in the intensive therapy unit (ITU) and not in a ward-based setting. For GDFT to be beneficial in acute pancreatitis, which involves a cascade of inflammatory events, therapy is likely to be most beneficial if commenced at the earliest opportunity to intervene following the onset of pancreatitis which would equate with the time of admission to hospital. There has been one randomised controlled trial on ward based GDFT in patients with acute pancreatitis comparing the effect of GDFT with Lactated ringers to normal saline on inflammatory response (14). However, the trial was based on optimisation of blood urea nitrogen (BUN) and not cardiac output parameters and failed to show a reduction in the inflammatory response or improved clinical outcomes. Whilst other endpoints such as heart rate (HR), urine output (UO), haematocrit levels and central venous pressures have been suggested for fluid therapy in acute pancreatitis, it is the optimisation of intravascular volume with fluid therapy guided by cardiac output measures that has been previously shown to be effective in decreasing complications and mortality in major surgery (15,16). There is currently no RCT investigating

For peer review only

the role of ward based GDFT using cardiac output parameters as target in patients with acute pancreatitis.

With the development of non-invasive cardiac output monitors such as Cheetah NICOM™ (Cheetah Medical Ltd, Maidenhead, Berkshire, UK), it is possible to measure the cardiac output in a ward setting (17). Ward-based GDFT has the potential to decrease the early inflammatory response and hence complications related to acute pancreatitis which might result in decreased mortality and improved health-related quality of life (HRQoL). Reduced inflammation would also lead to a reduced intensive care unit (ICU) and hospital stay providing significant cost savings to the NHS, employers and care providers. This two-centre RCT will aim to assess the feasibility of guiding the initial 48 hours of IV fluid administration in patients with acute pancreatitis using ward-based GDFT.

Methods

Study design and setting

The GAP trial has been designed in accordance with the SPIRIT guidelines as a two centre, feasibility RCT (18). The study will primarily investigate the ability to recruit patients at the selected sites to a feasibility RCT of ward-based GDFT versus standard fluid therapy in patients with acute pancreatitis, the rate of withdrawal from GDFT protocol, and the reasons for withdrawal from GDFT protocol. We will also assess the safety and practicality of ward-based GDFT and collect outcome measures which can be evaluated as end points for a subsequent multi-centre study on efficacy. Indicative costs will be collected to inform a subsequent cost effectiveness study. The trial outline is illustrated in Figure 1 and the schedule of enrolment, interventions and assessments is shown in Table 1.

Participants

For peer review only

Adults (>16 years) admitted as an emergency with acute pancreatitis will be included. Acute pancreatitis must be confirmed by the international consensus criteria (table 2) (19). The exclusion criteria will be patients transferred for the management of complications of acute pancreatitis, requiring immediate admission to the ICU, chronic pancreatitis in whom an acute exacerbation cannot be confirmed, a history of cardiac failure in the past three months and those unable to provide fully informed consent.

Recruitment

Patients referred from the emergency department for admission through the emergency surgical team will be screened by the general surgical registrar on-call. Due to the time-dependant nature of this trial, similar to most trauma and emergency surgery studies, a summarised and abbreviated patient information sheet (PIS) will be provided for patients initially on arrival and they will be subsequently provided with an in-depth version. Informed consent will be obtained by a member of the clinical or research team trained in Good Clinical Practice (GCP) (20). The trial nursing staff responsible for instituting the trial intervention will be contacted within four hours of diagnosis. Intervention or standard of care will commence within six hours of the diagnosis for the next 48 hours of inpatient stay. Since the primary goal of this study is to assess the feasibility of delivering *ward-based* GDFT, patients requiring escalation of care and admission to ICU during the intervention will only have continued cardiac output monitoring until completion of the 48 hour period.

Randomisation

This will be performed using ‘Sealed Envelope’, an internet-based randomisation system (www.sealedenvolpe.com). Eligible patients who have consented to take part in the trial will be 1:1 randomised to ward based GDFT or standard care, stratified by site on admission, prior to ward transfer. The trial research nurse responsible (unblinded) will log into the database and create randomisation codes for both groups online. Information on randomisation will be stored in the case report form (CRF).

For peer review only - http://bmjopen.bmj.com/site/about/guidelines.xhtml

Intervention

GDFT will be carried out for 48 hours. It can take up to 48 hours for the severity of pancreatitis to manifest and is considered the ‘golden’ period for interventions that may decrease severity (21). GDFT will be based on a standard algorithm which uses the stroke volume (SV) derived from non-invasive cardiac output monitoring using the Cheetah NICOM™ (figure 2). The fluid administration regimen will be as follows: maintenance fluid should be administered at 1.5 ml/kg/hr (based on ideal body weight) using a balanced crystalloid solution to be ensure that a regulated volume of fluid was given per hour in order to avoid fluid overload in this group. Previous studies have been criticised for excessive maintenance fluid prescribed in the GDFT group (22). It was also felt that placing restrictions on the maintenance fluid prescription may influence decision making in this group and affect the enthusiasm for clinicians to agree in patients participating in the study.

On admission SV is recorded and an initial bolus of 250ml of IV fluid (balanced electrolyte solution) is given over 5 to 10 minutes. If there is a sustained rise in SV of greater than 10% for 15 minutes or more, this indicates fluid responsiveness and a repeat 250ml bolus will be given. If there is not a rise in SV of greater than 10% then the patient is deemed fluid unresponsive and no further fluid boluses are administered. SV monitoring continues four hourly and if it decreases by more than 10% a further fluid bolus is administered as above.

Control (standard care group)

The choice of fluid type, volume and rate of administration on the first 48 hours of admission for patients randomised to the control arm will be decided by the clinical team caring for the patient in order to mimic a real-world situation. Cardiac output monitoring variables will be measured every 4 hours on the standard care arm during the first 48 hours, however, the results will be blinded to the clinical team. The choice of fluid type, volume and rate will be

For peer review only

recorded in detail. All other clinical, biochemical and HRQoL outcome measures will also be recorded for standard of care patients as outlined below in 'outcome measures' section.

The Cheetah NICOM™ device (Cheetah Medical Ltd, Maidenhead, Berkshire, UK) is a CE marked device has been purchased for this study and training has been arranged for all staff on the equipment and the associated fluid administration algorithm which will be a key component of the trial. The research team will follow hospital policy on signals for ICU outreach reviews and escalation of care to ICU. NICOM monitoring and outcome measures will be recorded in ICU for both control and GDFT arms.

Blinding

It will not be possible to blind the research or clinical team during the first 48 hours of the study. However, the participants, outcome assessors of health-related quality of life, and statisticians will be blinded to the groups. Patient blinding will be aided by NICOM monitoring of both intervention and control groups but performing GDFT in the intervention group alone. NICOM data from the control group will be collected at the same time points as the intervention group and will not be available to the treating clinicians.

Outcome measures

The primary outcome of the trial will be an assessment of feasibility. In particular, the ability to identify and recruit patients at the selected sites to a study of acute pancreatitis. A recruitment rate of at least 30% over 17-month trial period will be deemed as successful. We will also assess the availability of study team for a condition presenting as an emergency, ability to randomise and commence ward GDFT within 6 hours of admission, completion rate of 48 hours of GDFT, rate of withdrawal (less than 20%) from GDFT protocol, reasons for withdrawal from GDFT protocol and proportion of complications in the two groups. Although the study is not powered to show differences in haemodynamic variables, we will analyse the haemodynamic data (such as SV and CO) at the end of the trial for patients in both groups to,

For peer review only - http://bmjopen.bmj.com/site/about/guidelines.xhtml

at least, be able to conclude that we have optimised SV status of patients correctly according to the GDFT protocol drawn at the start of the trial. This data will be presented along with the main feasibility end-points.

Several secondary clinical, surgical, biochemical, safety and quality of life outcomes will also be collected. Mortality will be assessed as well. Clinical outcomes will include severity of pancreatitis as assessed by Glasgow score, proportion of patients with severe acute pancreatitis, necrotising pancreatitis, infected pancreatic necrosis, requiring ITU stay, requiring renal replacement therapy, requiring ventilation, requiring surgical interventions for complications related to pancreatitis and incidence of positive blood cultures. Resource use data for health economic analysis on length of hospital stay, length of ICU stay, and number of days ventilated, time to return to pre-pancreatitis activities, number of work days lost (in those who work), and costs (NHS and personal social services (PSS) perspectives) will be collected. Health-related quality of life (HRQoL) will be assessed using the well validated EQ5D questionnaire on admission as baseline and subsequently on day 7, day 30 and day 90. All complications of pancreatitis including treatment-related adverse events (post-radiological and surgical complications as classified by Clavien-Dindo classification) and serious adverse events related to intervention will be recorded up to discharge and at follow-up. Routine biochemical tests performed as part of clinical care including blood gases, liver function tests, clotting profile, renal function, amylase, full blood count and C-reactive protein (CRP) will be recorded on admission and daily up to two weeks and bi-weekly up to discharge. In addition, serum samples will be collected from participants in both groups on admission, 12, 24- and 48-hour time points and stored at -80 degrees for future analysis of biomarkers in acute pancreatitis.

Follow-up

For peer review only

267 Participants will be followed for 90 days. All clinical and HRQoL outcomes will be measured up to discharge and specifically at 30 days and 90 days by face-to-face or telephone follow-up. CRP will be measured at 24 hours and 48 hours and every 24 hours during the hospital stay as per routine clinical practice. Clinical outcomes on follow-up include cumulative hospital and intensive care unit stay including re-admissions, pre-defined complications including organ failure, sever pancreatitis, infected pancreatic necrosis, surgical interventions, development of sepsis, pulmonary oedema or acute respiratory distress syndrome as well as mortality. Resource use data including medications, planned and unplanned tests and procedures and use of allied healthcare professionals (e.g. physiotherapy, occupational therapy). HRQoL will be assessed using a validated questionnaire (EQ-5D).

Sample size calculation

Formal sample size calculations are not appropriate for a feasibility study, hence a convenient sample size of 50 patients, randomised equally to the two groups, has been chosen. Sample sizes between 24 and 50 have been recommended to estimate the standard deviation required for a sample size calculation for a subsequent large RCT aimed at evaluating the cost-effectiveness of GDFT (23).

Patient and Public Involvement

Patients involved in the trial preparation were surprised that there had been no progress in effective treatment for acute pancreatitis and felt that new therapeutic approaches were required. Some had experienced late complications and felt that better early treatment may prevent these late complications. The trial protocol was presented and discussed with patients to ensure that patient centred outcomes such as survival and quality of life were appropriately measured, and advice was sought on the conduct of the study. The burden of the intervention was assessed by patient representatives and was thought to be ethical and appropriate. The outcomes of the study will be disseminated to the participants after review and formulation by our study patient representative. We wish to thank our patient representatives for their contribution and participation in this process.

For peer review only

Trial management structure

A Trial Management Group (TMG) chaired by the Chief Investigator and comprising of leads at different sites, ICU research nurses, the surgical team representative, research fellows, statistician, qualitative researchers and health economist will meet fortnightly to discuss trial progress and address issues in recruitment and delivering the intervention. Trial progress will be reported to independent Data Monitoring Committee (DMC) comprising of experts in acute pancreatitis, medical statistics and clinical trials every 6 months.

All serious complications and any mortalities will be immediately referred to the sponsor and reported to the trial management group and independent data monitoring committee. The trial will be stopped in case of two or more treatment related suspected unexpected serious adverse reactions (SUSARs),

Data collection and analysis

Recruitment data which includes number of eligible patients presenting to the emergency department, number of patients screened, number of patients consenting, and number of patients randomised is recorded in a recruitment log in the emergency department. A CONSORT diagram will be presented to provide a detailed description of participant numbers at each time point during the trial (24). A paper CRF (Case Report Form) is created for each participant enrolled into the study. The data collected on the paper CRF is then transferred to a secure online database using the RedCap (Research Electronic Data Capture) platform.

Since this is a feasibility study, all analyses other than recruitment rate and withdrawal rates should be considered exploratory. The two groups will be compared to ensure they have similar baseline characteristics (table 3) using means and standard deviations or medians and inter-quartile ranges for continuous variables, as appropriate, and frequency counts and percentages for categorical variables.

For peer review only

For the primary outcome, i.e., feasibility, the proportion of patients who consent to be randomised and the rate of withdrawal from GDFT protocol will be presented with a 95% confidence interval. The median number of complications, graded by the Clavien-Dindo classification, in each group will be presented. The proportions of people with complications between the two groups will be presented and compared using appropriate statistical test and 95% confidence interval.

For the secondary outcomes, amongst patients who participate in the trial, all clinical and surgical outcome measures will be presented for each group separately using the mean, standard deviation, median, minimum and maximum for continuous outcomes and using frequencies and proportions for categorical variables.

Quality of life will also be summarised for each group using mean profile plots over time. The mean difference in quality of life scores between the two groups at 7 days up will be presented with a 95% confidence interval.

Kaplan-Meier survival estimates and plots will be presented to compare survival rates between the two groups.

All other secondary outcomes collected over time will be summarised for each group using mean profile plots. Mean differences for continuous outcomes and difference in proportion for binary outcomes shall be presented, with appropriate 95% confidence intervals, at 30 and 90 days.

The number and nature of adverse events shall be reported for each group. No formal comparisons between the groups will be made and no hypothesis tests will be carried out. The results will inform us how sensitive the outcome measures are and, along with other

For peer review only

information, will be used to determine the primary outcome of a subsequent large RCT. The results will also inform a sample size calculation for the primary outcome chosen.

Assessment of feasibility

This is a feasibility study and one aspect of this feasibility evaluation is the ability to recruit patients with acute pancreatitis into a trial of this nature. According to hospital episode statistics, an in-house review of admissions for acute pancreatitis to the Royal Free Hospital over the last three years showed 107 cases per year. After excluding patients who were transferred from another hospital for tertiary care, those who required immediate ICU admission, those with chronic pancreatitis in whom an acute exacerbation could not be confirmed by internal consensus criteria, current or past cardiac failure, or unable to provide fully informed consent, 80 patients per year would be eligible for this trial from a single centre. With a second centre we would anticipate 120 suitable patients per year. We anticipate recruiting at least 30% of potential participants which equates to 36 participants annually and a recruitment time of 17 months to recruit the 50 patients.

For feasibility assessment, a recruitment log will be used to collect information on suitability and consent rate as well as availability of trials team for GDFT, consent, failure to commence ward GDFT, withdrawal with reasons and failure to complete the study. In case of lower than anticipated recruitment at six months, we will perform the following; a) analyse the results of the qualitative research earlier and identify the reasons for poor recruitment (and take actions to resolve the problems) if we get the anticipated number of patients screened but the recruitment rate is lower than anticipated, b) recruitment of additional centres if we do not get the anticipated number of potential participants screened but the recruitment rate is at least as good as the anticipated rate c) a combination of the above if the number of potential participants screened and the recruitment rate are lower than anticipated.

For peer review only

We will explore the reasons for participation and non-participation of eligible patients and patients' and clinicians' acceptability of the trial to assist in optimisation of recruitment strategies employed for the definitive trial. Non-participation can be related to how the clinical trial is presented to the patient, and how the patient assimilates this information. It is therefore important to understand how patients perceive information about potential participation and their experiences of receiving information relating to the trial. Interviews with a sample of eligible patients will explore patient perspectives of treatment, their understanding of the two treatments, reasons for taking part or refusing the trial, and the acceptability of randomisation between the procedures. Interviews with clinical staff will explore their views about the trial, clinical equipoise, and their understanding of the recruitment challenges. Semi-structured interviews will be informed by a topic guide developed in conjunction with the trial management group which includes patient representatives. Patient information sessions (recruiter meetings) will be audio recorded to examine how information is presented, and identify issues potentially affecting trial recruitment. Patient information sessions will be analysed at an early stage if there is poor recruitment and will inform the development of any additional training materials for recruitment for the definitive trial.

Progress to full trial

The criteria to progress to a subsequent full trial will be determined quantitatively as a) consent rate of at least 30%, the ability to recruit 50 patients to the study at the two sites over 17 months, b) GDFT can be successfully performed within 6 hours of diagnosis of acute pancreatitis and can be continued until at least 48 hours after admission in a minimum of 80% of participants randomised to GDFT, and c) the complication rate in the intervention group is not more than 10% higher than that of the control group at 90 days. A cut off of 30% has been chosen for recruitment as this would be an achievable target in the definitive trial. A lower recruitment rate may indicate a lack of acceptability among clinicians and/or patients to participate in the definitive trial. In addition, a recruitment rate less than 30% is likely to make the subsequent definitive trial very expensive. A less than 30% recruitment may also highlight

For peer review only

406 issues related to generalisability of the results, indicating a reduction in value of the
407 subsequent definitive trial in terms of applicability in NHS.
408
409 *Ethics and Dissemination*
410 The trial protocol (v2) has been reviewed and approved by the London Central Research
411 Ethics Committee (REC ref: 17/LO/1235, project ID: 221872). The results of this study will
412 be presented to the national and international meetings with interest in the management of
413 acute pancreatitis and prepared for publication in peer-reviewed journals with a readership in
414 general surgery and critical care.
415
416 **Discussion**
417 Acute pancreatitis is an inflammatory disease associated with a high mortality rate (25).
418 Adequate fluid resuscitation has been identified as a key intervention in prevention of systemic
419 complications, however, the debate over the type, rate, or amount of intravenous fluids to be
420 administered is ongoing (26). With the advent of non-invasive cardiac output monitoring
421 (NICOM) devices, delivering haemodynamic guided fluid replacement is now possible in
422 patients admitted to the general ward. The GAP trial is the first ward-based RCT of GDFT in
423 acute pancreatitis. The trial will assess feasibility and safety of delivering this intervention in
424 the ward and if successful, will progress to a multicentre trial to assess efficacy and cost-
425 effectiveness.
426
427 **Trial Status**
428 The GAP trial opened for recruitment on 8th January 2018 for a period of 18 months.
429
430 **List of Abbreviations**
431 - C-Reactive Protein (CRP)
432 - Data Monitoring Committee (DMC)
433 - GDFT in Acute Pancreatitis (GAP)

For peer review only

- Goal Directed Fluid Therapy (GDFT)
- Health-Related Quality of Life (HRQoL)
- Intensive Care Unit (ICU)
- Intravenous (IV)
- National Health System (NHS)
- Non-Invasive Cardiac Output Monitor (NICOM)
- Patient Information Sheet (PIS)
- Personal Social Services (PSS)
- Randomized Control Trial (RCT)
- Stroke Volume (SV)
- Suspected Unexpected Serious Adverse Reactions (SUSAR)
- Trial Management Group (TMG)

Declarations

Ethical approval and consent to participate

The trial protocol (v2) has been reviewed and approved by the London Central Research Ethics Committee (REC ref: 17/LO/1235, project ID: 221872). Informed consent will be obtained from eligible patients after screening by a member of the research or clinical team trained in Good Clinical Practice (GCP). The trial has been registered on ISRCTN (ISRCTN 36077283) on 09 April 2018 (http://www.isrctn.com/ISRCTN36077283).

Consent for publication

Not applicable.

Availability of data and materials

Data from this trial will be made available after analysis and publication upon reasonable request from the corresponding author.

For peer review only

Competing interests

The authors declare that they have no competing interests.

Funding

This study is funded by the National Institute for Health Research (NIHR) Research for Patient Benefit Programme (RfPB) (Grant Reference Number PB-PG-0815-20002). This paper presents independent research funded by the (NIHR) under its Research for Patient Benefit (RfPB) Programme (Grant Reference Number PB-PG-0815-20002). The views expressed are those of the author(s) and not necessarily those of the NHS, the NIHR or the Department of Health.

For peer review only

Author contributions

FF manuscript preparation, mechanistic components of protocol design, trial and database management, FS manuscript preparation, trial management, FR statistical analysis plan, KG major contribution to design and funding application, DM protocol design of GDFT regimes, trial management, SS initial protocol developments and ethical approvals, CE, MC, OS, GA, MMcN and HF trial research nurses trained in delivering GDFT, data collection, JS health economic analysis design, MV participant enrolment planning through emergency surgery , BD principal investigator and grant holder, overall trial management, study design and review of manuscript. All authors read and approved the final manuscript.

Acknowledgements

We are grateful for the support of our emergency surgical colleagues Mr Olegunju Ogunbiyi, Mr Jonathan Knowles, Mr Rovan D'Souza, Mr Colin Hart, Mr Ahmad Hamad, the surgical registrars Ms Tanzeela Gala, Ms Karen Bosch, Ms Aliza Abeles, Mr Guillaume Lafaurie, Mr Tom Hanna, Mr Stephanos Pericleous, our emergency department lead Dr Jonathan Costello, without whom the conduct of this trial would not be possible. We wish to thank our patient

For peer review only - http://bmjopen.bmj.com/site/about/guidelines.xhtml

representative Ms Lucy Cotterell for their contributions. We gratefully acknowledge the financial support of the Wellington hospital, London, in their HPB Surgery Fellowship funding for Mr Farid Froghi.

References

1. Roberts SE, Akbari A, Thorne K, Atkinson M, Evans PA. The incidence of acute pancreatitis: Impact of social deprivation, alcohol consumption, seasonal and demographic factors. Aliment Pharmacol Ther [Internet]. 2013 Sep [cited 2018 Sep 5];38(5):539–48. Available from: http://www.ncbi.nlm.nih.gov/pubmed/23859492
2. Johnson CD. UK guidelines for the management of acute pancreatitis [Internet]. Vol. 54, Gut. 2005. p. iii1–9. Available from: http://gut.bmj.com/content/54/suppl_3/iii1
3. Párniczky A, Kui B, Szentesi A, Balázs A, Szûcs Á, Mosztbacher D, et al. Prospective, Multicentre, Nationwide Clinical Data from 600 Cases of Acute Pancreatitis. PLoS One. 2016;11(10):1–19.
4. Leung PS, Ip SP. Pancreatic acinar cell: Its role in acute pancreatitis. Vol. 38, International Journal of Biochemistry and Cell Biology. 2006. p. 1024–30.
5. Andersson R, Andersson B, Andersson E, Axelsson J, Eckerwall G, Tingstedt B. Acute pancreatitis - From cellular signalling to complicated clinical course. Hpb. 2007;9(6):414–20.
6. 'tHart NA, Leuvenink HGD, Ploeg RJ. New solutions in organ preservation. Transplant Rev [Internet]. 2002 Jul 1 [cited 2017 Dec 11];16(3):131–41. Available from: http://www.sciencedirect.com/science/article/pii/S0955470X02800027
7. De Beaux AC, Palmer KR, Carter DC. Factors influencing morbidity and mortality in acute pancreatitis; an analysis of 279 cases. Gut [Internet]. 1995 Jul [cited 2018 Sep 14];37(1):121–6. Available from: http://www.ncbi.nlm.nih.gov/pubmed/7672660
8. Vege SS, Gardner TB, Chari ST, Munukuti P, Pearson RK, Clain JE, et al. Low Mortality and High Morbidity in Severe Acute Pancreatitis Without Organ Failure: A Case for Revising the Atlanta Classification to Include "Moderately Severe Acute

For peer review only

Pancreatitis." Am J Gastroenterol [Internet]. 2009 Mar 3 [cited 2018 Sep 14];104(3):710–5. Available from: http://www.nature.com/articles/ajg200877

9. Moggia E, Koti R, Belgaumkar AP, Fazio F, Pereira SP, Davidson BR, et al. Pharmacological interventions for acute pancreatitis. Vol. 2017, Cochrane Database of Systematic Reviews. 2017. p. 1–14.

10. Iqbal U, Anwar H, Scribani M. Ringer's Lactate vs Normal Saline in Acute Pancreatitis: A Systematic Review and meta- analysis.

11. Szentesi A, Tóth E, Bálint E, Fanczal J, Madácsy T, Laczkó D, et al. Analysis of research activity in gastroenterology: Pancreatitis is in real danger. PLoS One [Internet]. 2016 [cited 2019 Apr 11];11(10):e0165244. Available from: http://www.ncbi.nlm.nih.gov/pubmed/27776171

12. Pearse RM, Harrison DA, MacDonald N, Gillies MA, Blunt M, Ackland G, et al. Effect of a perioperative, cardiac output-guided hemodynamic therapy algorithm on outcomes following major gastrointestinal surgery a randomized clinical trial and systematic review. JAMA - J Am Med Assoc [Internet]. 2014 Jun 4 [cited 2018 Oct 11];311(21):2181–90. Available from: http://jama.jamanetwork.com/article.aspx?doi=10.1001/jama.2014.5305

13. Hamilton MA, Cecconi M, Rhodes A. A Systematic Review and Meta-Analysis on the Use of Preemptive Hemodynamic Intervention to Improve Postoperative Outcomes in Moderate and High-Risk Surgical Patients. Anesth Analg [Internet]. 2011 Jun [cited 2016 Aug 27];112(6):1392–402. Available from: http://content.wkhealth.com/linkback/openurl?sid=WKPTLP:landingpage&an=00000539-201106000-00027

14. Wu BU, Hwang JQ, Gardner TH, Repas K, Delee R, Yu S, et al. Lactated Ringer's Solution Reduces Systemic Inflammation Compared With Saline in Patients With Acute Pancreatitis. Clin Gastroenterol Hepatol. 2011;9(8):710–7.

15. Rivers EP, Ander DS, Powell D. Central venous oxygen saturation monitoring in the critically ill patient. Curr Opin Crit Care. 2001;7(3):204–11.

For peer review only

16. Bortolotti P, Saulnier F, Colling D, Redheuil A, Preau S. New tools for optimizing fluid resuscitation in acute pancreatitis. World J Gastroenterol [Internet]. 2014 Nov 21 [cited 2018 Oct 11];20(43):16113–22. Available from: http://www.ncbi.nlm.nih.gov/pubmed/25473163

17. Dunham CM, Chirichella TJ, Gruber BS, Ferrari JP, Martin JA, Luchs BA, et al. Emergency department noninvasive (NICOM) cardiac outputs are associated with trauma activation, patient injury severity and host conditions and mortality. J Trauma Acute Care Surg. 2012;73(2):479–85.

18. Chan A-W, Tetzlaff JM, Gøtzsche PC, Altman DG, Mann H, Berlin JA, et al. SPIRIT 2013 explanation and elaboration: guidance for protocols of clinical trials. BMJ [Internet]. 2013 Jan 8 [cited 2016 Oct 25];346:e7586. Available from: http://www.ncbi.nlm.nih.gov/pubmed/23303884

19. Tenner S, Baillie J, Dewitt J, Vege SS. American college of gastroenterology guideline: Management of acute pancreatitis. Am J Gastroenterol. 2013;108(9):1400–15.

20. Good Clinical Practice (GCP) [Internet]. [cited 2018 Sep 14]. Available from: https://www.nihr.ac.uk/our-faculty/clinical-research-staff/learning-and-development/national-directory/good-clinical-practice/

21. Fisher JM, Gardner TB. The “Golden Hours” of Management in Acute Pancreatitis. Am J Gastroenterol [Internet]. 2012 Aug 3 [cited 2018 Oct 11];107(8):1146–50. Available from: http://www.nature.com/articles/ajg201291

22. Challand C, Struthers R, Sneyd JR, Erasmus PD, Mellor N, Hosie KB, et al. Randomized controlled trial of intraoperative goal-directed fluid therapy in aerobically fit and unfit patients having major colorectal surgery. Br J Anaesth [Internet]. 2012 Jan [cited 2016 Oct 10];108(1):53–62. Available from: http://www.ncbi.nlm.nih.gov/pubmed/21873370

23. Sim J, Lewis M. The size of a pilot study for a clinical trial should be calculated in relation to considerations of precision and efficiency. J Clin Epidemiol [Internet]. 2012

For peer review only

Mar 1 [cited 2018 Sep 15];65(3):301–8. Available from: http://www.ncbi.nlm.nih.gov/pubmed/22169081

24. Moher D, Schulz KF, Altman DG. The CONSORT statement: revised recommendations for improving the quality of reports of parallel-group randomised trials. Lancet [Internet]. 2001 Apr 14 [cited 2018 Dec 22];357(9263):1191–4. Available from: https://www.sciencedirect.com/science/article/pii/S0140673600043373?via%3Dihub

25. Bank S, Singh P, Pooran N, Stark B. Evaluation of Factors That Have Reduced Mortality From Acute Pancreatitis Over the Past 20 Years. J Clin Gastroenterol [Internet]. 2002;35(1). Available from: https://journals.lww.com/jcge/Fulltext/2002/07000/Evaluation_of_Factors_That_Have_Reduced_Mortality.12.aspx

26. Aggarwal A, Manrai M, Kochhar R. Fluid resuscitation in acute pancreatitis. World J Gastroenterol [Internet]. 2014 Dec 28 [cited 2018 Sep 15];20(48):18092–103. Available from: http://www.ncbi.nlm.nih.gov/pubmed/25561779

For peer review only

Table 1. Schedule of enrolment, interventions and assessments

Procedures	Enrolment	Allocation	Post-allocation								Close-out
**Time point**	**-t0**	**0**	**0 hrs**	**12 hrs**	**24 hrs**	**48 hrs**	**discharge**	**7 days**	**30 days**	**90 days**	
**ENROLMENT**											
Eligibility screen	x										
PIS given	x										
Informed consent	x										
Registration	x										
Allocation		x									
**INTERVENTION**											
GDFT (intervention)			◆—	—	—	—◆					
Standard care (control)			◆—	—	—	—◆					
ASSESSMENTS											
NICOM Monitoring			◆—	—	—	—◆					
Blood tests			x	x	x	x					
HRQoL			x					x	x	x	x
Complications			x	x	x	x	x		x	x	x
Other outcomes			x	x	x	x	x		x	x	x

For peer review only

Table 2. International consensus criteria for acute pancreatitis

Acute pancreatitis diagnosis confirmed with two of the following three features:
• Abdominal pain consistent with acute pancreatitis (acute onset of a persistent, severe, epigastric pain often radiating to the back)<br>• Serum amylase or lipase activity at least three times greater than the upper limit of normal.<br>• Characteristic findings of acute pancreatitis on contrast-enhanced computed tomography (CECT) and less commonly magnetic resonance imaging (MRI) or transabdominal ultrasonography.

For peer review only

Table 3. Baseline characteristics of patients

Age
Sex
Ethnicity
BMI
Time since onset of symptoms
Presumed cause of pancreatitis
Comorbidities
Baseline observations
Baseline renal function
Baseline blood gas parameters
Glasgow severity score on admission
Intravenous fluids prior to intervention

For peer review only

Figure Legends:

Figure 1. Trial flow diagram
Patients are screened by the medical and surgical team in the emergency department for eligibility and referred for consent and registration. The trial nurse or surgical team provide trial patient information sheets (abbreviated and full versions) and gain informed consent. Trial nurses then randomise the patient to either GDFT or standard care and commence NICOM monitoring for 48 hours. Follow-up will be at the point of discharge, 30 days and 90 days.

Figure 2. Goal-directed fluid therapy (intervention) protocol
Flow diagram of SV optimisation with maintenance fluid administration. Patients randomised to GDFT will receive a 1.5ml/kg/hr of intravenous crystalloid maintenance fluid and four hourly visits from the trial nurses. At the first 4-hour visit, a bolus of 250mL crystalloid over 5-10mins will be administered. A SV rise of >10% would indicate fluid responsiveness and a further cycle of fluid bolus is administered until there is no SV rise >10%. At the next 4 hourly visit if SV has dropped by >10% the cycle is repeated.

For peer review only

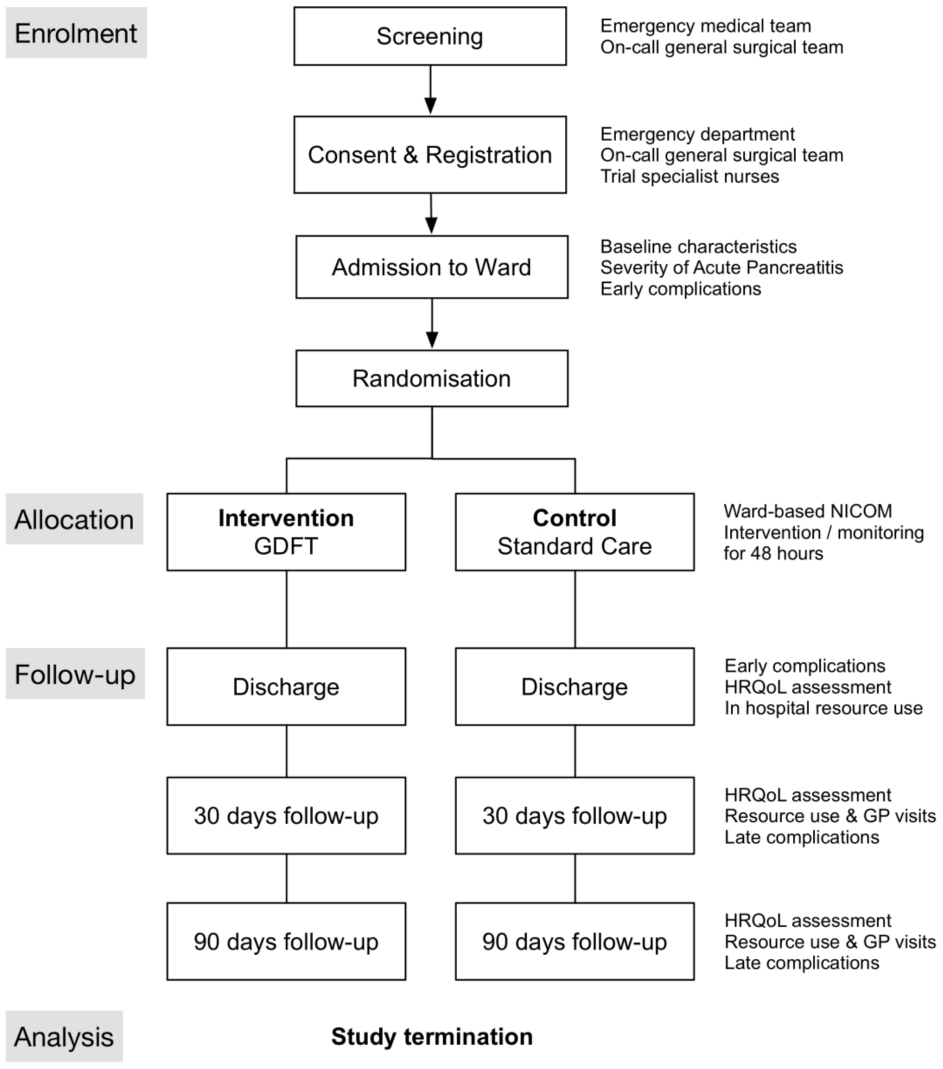

Figure 1 Trial flow diagram

207x234mm (300 x 300 DPI)

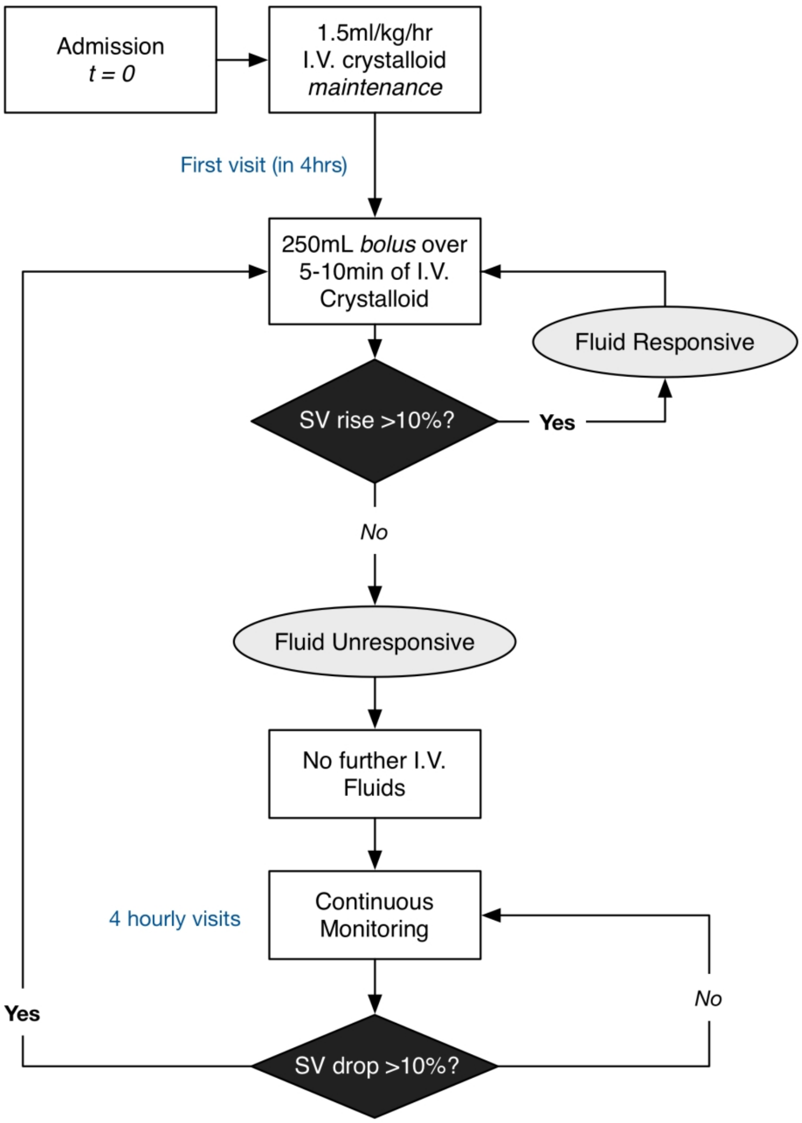

Figure 2 Goal-directed fluid therapy protocol

155x216mm (300 x 300 DPI)

Reporting checklist for protocol of a clinical trial.

Based on the SPIRIT guidelines.

Instructions to authors

Complete this checklist by entering the page numbers from your manuscript where readers will find each of the items listed below.

Your article may not currently address all the items on the checklist. Please modify your text to include the missing information. If you are certain that an item does not apply, please write "n/a" and provide a short explanation.

Upload your completed checklist as an extra file when you submit to a journal.

In your methods section, say that you used the SPIRIT reporting guidelines, and cite them as:

Chan A-W, Tetzlaff JM, Altman DG, Laupacis A, Gøtzsche PC, Krleža-Jerić K, Hróbjartsson A, Mann H, Dickersin K, Berlin J, Doré C, Parulekar W, Summerskill W, Groves T, Schulz K, Sox H, Rockhold FW, Rennie D, Moher D. SPIRIT 2013 Statement: Defining standard protocol items for clinical trials. Ann Intern Med. 2013;158(3):200-207

		Reporting Item	Page Number
Title	#1	Descriptive title identifying the study design, population, interventions, and, if applicable, trial acronym	1
Trial registration	#2a	Trial identifier and registry name. If not yet registered, name of intended registry	2
Trial registration: data set	#2b	All items from the World Health Organization Trial Registration Data Set	2
Protocol version	#3	Date and version identifier	16
Funding	#4	Sources and types of financial, material, and other support	17
Roles and responsibilities: contributorship	#5a	Names, affiliations, and roles of protocol contributors	17
Roles and responsibilities:	#5b	Name and contact information for the trial sponsor	17

For peer review only

sponsor contact information			
Roles and responsibilities: sponsor and funder	#5c	Role of study sponsor and funders, if any, in study design; collection, management, analysis, and interpretation of data; writing of the report; and the decision to submit the report for publication, including whether they will have ultimate authority over any of these activities	17
Roles and responsibilities: committees	#5d	Composition, roles, and responsibilities of the coordinating centre, steering committee, endpoint adjudication committee, data management team, and other individuals or groups overseeing the trial, if applicable (see Item 21a for data monitoring committee)	17, 10
Background and rationale	#6a	Description of research question and justification for undertaking the trial, including summary of relevant studies (published and unpublished) examining benefits and harms for each intervention	4-6
Background and rationale: choice of comparators	#6b	Explanation for choice of comparators	4-6 / 8
Objectives	#7	Specific objectives or hypotheses	6
Trial design	#8	Description of trial design including type of trial (eg, parallel group, crossover, factorial, single group), allocation ratio, and framework (eg, superiority, equivalence, non-inferiority, exploratory)	6
Study setting	#9	Description of study settings (eg, community clinic, academic hospital) and list of countries where data will be collected. Reference to where list of study sites can be obtained	6
Eligibility criteria	#10	Inclusion and exclusion criteria for participants. If applicable, eligibility criteria for study centres and individuals who will perform the interventions (eg, surgeons, psychotherapists)	6
Interventions: description	#11a	Interventions for each group with sufficient detail to allow replication, including how and when they will be administered	8
Interventions: modifications	#11b	Criteria for discontinuing or modifying allocated interventions for a given trial participant (eg, drug dose change in response to harms, participant request, or improving / worsening disease)	8
Interventions:	#11c	Strategies to improve adherence to intervention protocols, and any	8

For peer review only

adherance		procedures for monitoring adherence (eg, drug tablet return; laboratory tests)	
Interventions: concomitant care	#11d	Relevant concomitant care and interventions that are permitted or prohibited during the trial	8
Outcomes	#12	Primary, secondary, and other outcomes, including the specific measurement variable (eg, systolic blood pressure), analysis metric (eg, change from baseline, final value, time to event), method of aggregation (eg, median, proportion), and time point for each outcome. Explanation of the clinical relevance of chosen efficacy and harm outcomes is strongly recommended	9
Participant timeline	#13	Time schedule of enrolment, interventions (including any run-ins and washouts), assessments, and visits for participants. A schematic diagram is highly recommended (see Figure)	Table 1
Sample size	#14	Estimated number of participants needed to achieve study objectives and how it was determined, including clinical and statistical assumptions supporting any sample size calculations	10
Recruitment	#15	Strategies for achieving adequate participant enrolment to reach target sample size	7
Allocation: sequence generation	#16a	Method of generating the allocation sequence (eg, computer-generated random numbers), and list of any factors for stratification. To reduce predictability of a random sequence, details of any planned restriction (eg, blocking) should be provided in a separate document that is unavailable to those who enrol participants or assign interventions	7
Allocation concealment mechanism	#16b	Mechanism of implementing the allocation sequence (eg, central telephone; sequentially numbered, opaque, sealed envelopes), describing any steps to conceal the sequence until interventions are assigned	7
Allocation: implementation	#16c	Who will generate the allocation sequence, who will enrol participants, and who will assign participants to interventions	7
Blinding (masking)	#17a	Who will be blinded after assignment to interventions (eg, trial participants, care providers, outcome assessors, data analysts), and how	9
Blinding (masking):	#17b	If blinded, circumstances under which unblinding is permissible,	9

For peer review only

emergency unblinding		and procedure for revealing a participant's allocated intervention during the trial	
Data collection plan	#18a	Plans for assessment and collection of outcome, baseline, and other trial data, including any related processes to promote data quality (eg, duplicate measurements, training of assessors) and a description of study instruments (eg, questionnaires, laboratory tests) along with their reliability and validity, if known. Reference to where data collection forms can be found, if not in the protocol	11
Data collection plan: retention	#18b	Plans to promote participant retention and complete follow-up, including list of any outcome data to be collected for participants who discontinue or deviate from intervention protocols	11
Data management	#19	Plans for data entry, coding, security, and storage, including any related processes to promote data quality (eg, double data entry; range checks for data values). Reference to where details of data management procedures can be found, if not in the protocol	11
Statistics: outcomes	#20a	Statistical methods for analysing primary and secondary outcomes. Reference to where other details of the statistical analysis plan can be found, if not in the protocol	11-13
Statistics: additional analyses	#20b	Methods for any additional analyses (eg, subgroup and adjusted analyses)	11
Statistics: analysis population and missing data	#20c	Definition of analysis population relating to protocol non-adherence (eg, as randomised analysis), and any statistical methods to handle missing data (eg, multiple imputation)	11
Data monitoring: formal committee	#21a	Composition of data monitoring committee (DMC); summary of its role and reporting structure; statement of whether it is independent from the sponsor and competing interests; and reference to where further details about its charter can be found, if not in the protocol. Alternatively, an explanation of why a DMC is not needed	
Data monitoring: interim analysis	#21b	Description of any interim analyses and stopping guidelines, including who will have access to these interim results and make the final decision to terminate the trial	10
Harms	#22	Plans for collecting, assessing, reporting, and managing solicited and spontaneously reported adverse events and other unintended effects of trial interventions or trial conduct	11

For peer review only

Auditing	#23	Frequency and procedures for auditing trial conduct, if any, and whether the process will be independent from investigators and the sponsor	11
Research ethics approval	#24	Plans for seeking research ethics committee / institutional review board (REC / IRB) approval	16
Protocol amendments	#25	Plans for communicating important protocol modifications (eg, changes to eligibility criteria, outcomes, analyses) to relevant parties (eg, investigators, REC / IRBs, trial participants, trial registries, journals, regulators)	11
Consent or assent	#26a	Who will obtain informed consent or assent from potential trial participants or authorised surrogates, and how (see Item 32)	N/A
Consent or assent: ancillary studies	#26b	Additional consent provisions for collection and use of participant data and biological specimens in ancillary studies, if applicable	N/A
Confidentiality	#27	How personal information about potential and enrolled participants will be collected, shared, and maintained in order to protect confidentiality before, during, and after the trial	11
Declaration of interests	#28	Financial and other competing interests for principal investigators for the overall trial and each study site	16
Data access	#29	Statement of who will have access to the final trial dataset, and disclosure of contractual agreements that limit such access for investigators	16
Ancillary and post trial care	#30	Provisions, if any, for ancillary and post-trial care, and for compensation to those who suffer harm from trial participation	N/A
Dissemination policy: trial results	#31a	Plans for investigators and sponsor to communicate trial results to participants, healthcare professionals, the public, and other relevant groups (eg, via publication, reporting in results databases, or other data sharing arrangements), including any publication restrictions	15
Dissemination policy: authorship	#31b	Authorship eligibility guidelines and any intended use of professional writers	N/A
Dissemination policy: reproducible research	#31c	Plans, if any, for granting public access to the full protocol, participant-level dataset, and statistical code	15
Informed consent	#32	Model consent form and other related documentation given to	Appendix

For peer review only

materials		participants and authorised surrogates	
Biological specimens	#33	Plans for collection, laboratory evaluation, and storage of biological specimens for genetic or molecular analysis in the current trial and for future use in ancillary studies, if applicable	10

The SPIRIT checklist is distributed under the terms of the Creative Commons Attribution License CC-BY-ND 3.0. This checklist can be completed online using https://www.goodreports.org/, a tool made by the EQUATOR Network in collaboration with Penelope.ai

For peer review only
